# Potential invasive plant expansion in global ecoregions under climate change

**DOI:** 10.7717/peerj.6479

**Published:** 2019-03-05

**Authors:** Chun-Jing Wang, Qiang-Feng Li, Ji-Zhong Wan

**Affiliations:** 1 State Key Laboratory of Plateau Ecology and Agriculture, Qinghai University, Xining, China; 2 College of Agriculture and Animal Husbandry, Qinghai University, Xining, China

**Keywords:** ISSG, Invasive plant species, Climate change, Terrestrial ecoregions, Species distribution modelling, Freshwater ecoregions

## Abstract

Climate change is increasing the risk of invasive plant expansion worldwide. However, few studies have specified the relationship between invasive plant expansion and ecoregions at the global scale under climate change. To address this gap, we provide risk maps highlighting the response of invasive plant species (IPS), with a focus on terrestrial and freshwater ecoregions to climate change, and further explore the climatic features of ecosystems with a high potential for invasive plant expansion under climate change. We use species distribution modelling to predict the suitable habitats of IPS with records at the global scale. Hotspots with a potential risk of IPS (such as aquatic plants, trees, and herbs) expanding in global ecoregions were distributed in Northern Europe, the UK, South America, North America, southwest China, and New Zealand. Temperature changes were related to the potential of IPS expansion in global ecoregions under climate change. Coastal and high latitude ecoregions, such as temperate forests, alpine vegetation, and coastal rivers, were severely infiltrated by IPS under climate change. Monitoring strategies should be defined for climate change for IPS, particularly for aquatic plants, trees, and herbs in the biomes of regions with coastal or high latitudes. The role of climate change on the potential for IPS expansion should be taken into consideration for biological conservation and risk evaluation of IPS at ecoregional scales.

## Introduction

Invasion by plant species is a serious threat to native and managed ecosystems under climate change (Hellmann et al., 2008; Bai et al., 2013; Sheppard, 2013; Early et al., 2016). Climate change has the potential to rearrange the ecologically suitable areas of a species and promote invasive plant species (IPS) to establish viable populations, allowing IPS to subsequently expand over large geographic areas (Hoffmann & Sgrò, 2011; Petitpierre et al., 2012; Bellard et al., 2013). This could drive IPS into areas with high protection values, such as nature reserves, biodiversity hotspots, and important ecoregions, causing negative economic and ecological impacts (Bradley, Oppenheimer & Wilcove, 2009; Beaumont et al., 2011; Richardson & Rejmánek, 2011; Vicente et al., 2013; Bellard et al., 2014). Knowledge of the impact of global climate change on IPS can promote plant invasion management around the world (Hellmann et al., 2008; Bai et al., 2013; Bellard et al., 2013). Invasion management can include monitoring, prevention, and control of IPS expansion (Hellmann et al., 2008; Miller et al., 2010; Beaumont et al., 2011; Kalusová et al., 2013). With the acceleration of globalisation and the rapid pace of climate change, the spread of IPS has become a global problem (Ehrenfeld, 2005). Of the 100 most invasive species of the world, belonging to many taxonomic groups from microorganisms to plants and vertebrates, 36 are IPS, which seriously threaten the surrounding natural ecosystems and even lead to social problems worldwide (Lowe et al., 2000). Changes in species composition have been found suggesting that IPS may grow faster than native species as a result of global changes (Vila & Weiner, 2004; Mortensen et al., 2009). For example, Polygonum cuspidatum can threaten plant diversity and natural ecosystems due to habitat disturbances (Mortensen et al., 2009), and the invasion of *Acacia mearnsii* can cause an actual economic loss in South Africa (Van Wilgen et al., 2011). Therefore, there is an urgent need to evaluate the expansion of IPS under climate change.

Previous studies have primarily focused on the expansion risk of a group of IPS at regional scales, or representative species including some IPS at the global scale (Bai et al., 2013; Bellard et al., 2013; Vicente et al., 2013). Ecoregions are designed to help users visualise and understand similarities across complex multivariate environmental factors by grouping areas into similar categories (Olson et al., 2001; Abell et al., 2008), and the delineation of ecoregions can promote biodiversity conservation across different spatial scales (Jenkins & Joppa, 2009; Beaumont et al., 2011; Bajer et al., 2016; Saura et al., 2017). Hence, the effectiveness of biodiversity protection in many ecoregions around the world may decrease due to the negative impacts of plant invasion on native and managed ecosystems (Thuiller et al., 2005; Vicente et al., 2013; McConnachie et al., 2015; Foxcroft et al., 2017; Wan & Wang, 2018). However, many ecoregions have been invaded by IPS (Richardson et al., 2000; Thuiller et al., 2005; Bellard et al., 2015; Foxcroft et al., 2017; Wan & Wang, 2018). For example, future climate change has a large potential to drive IPS into ecoregions that are highly valuable for the protection of biodiversity in South Africa and the eastern US; the abilities of some protected areas to conserve biodiversity may be affected by plant invasion in ecoregions under climate change (Bradley, Wilcove & Oppenheimer, 2010; Donaldson et al., 2014; McConnachie et al., 2015; Foxcroft et al., 2017). To decrease the invasion risk of IPS, we should assess the potential of invasive plant expansion in global ecoregions under climate change.

However, few studies have specified the relationship between IPS expansion and climate change in global ecoregions under climate change. Bellard et al. (2013) projected the distributions of 36 of the world’s worst IPS across different biomes and proposed some management suggestions for invasion prevention and control, however, the number of IPS investigated was limited in this study. To address this knowledge gap, IPS with a wide distribution range and maps of terrestrial and freshwater ecoregions should be utilised to evaluate the potential of IPS to expand in global ecoregions under climate change. Furthermore, assessment of the expansion risk of IPS at the global ecoregion level could provide an important theoretical basis for the prevention and control of IPS at a global scale (Thuiller et al., 2005; Bellard et al., 2013, 2014; Wan & Wang, 2018).

Climatic suitability modellings were used to assess the possibility of IPS expansion in global ecoregions under climate change based on climatic niche conservatism (Petitpierre et al., 2012; Wan, Wang & Yu, 2017). Climatic suitability modellings are powerful tools for predicting species distribution and thus support biological conservation and risk assessment of biological invasion (Thuiller et al., 2005). These modellings have used occurrence records of IPS and climatic factors to assess the distribution of IPS at large scales (Thuiller et al., 2005; Bellard et al., 2013, 2014). The use of climatic suitability modellings in biological invasion gives us the new insights into the prevention and control of IPS at ecoregional scales. Niche conservatism, as a key requirement, indicates that species tend to grow and survive under the same environmental conditions in native and invaded ranges (Wiens et al., 2010; Petitpierre et al., 2012). Similarity in the climate between native and target regions has long been recognised as a basic requirement for successful invasion (Stigall, 2014; Gillard et al., 2017). Thus, we need to attach importance to niche conservatism for plant species. Such a niche conservatism hypothesis indicates a stable climatic suitability of plant species between native and invasive regions (Thuiller et al., 2005; Elith et al., 2011; Petitpierre et al., 2012).

In this work, we evaluate the potential of IPS to expand in global ecoregions under climate change by focusing on two specific questions: (1) where are the regions with the potential for IPS expansion of terrestrial and freshwater ecoregions under climate change; and (2) what are the climatic features of ecoregions with high IPS potential expansion under climate change. To address these two questions, we first used Maxent, a common climatic suitability modelling approach (Phillips, Anderson & Schapire, 2006), to model the climatic suitability of IPS under climate change; second, we mapped the potential of IPS expansion under climate change. Next, we assessed the potential of IPS to expand in terrestrial and freshwater ecoregions based on ecoregion biomes and plant growth forms. Finally, based upon our results, we propose a strategy for invasion management.

## Materials and Methods

### Study areas

Data related to global terrestrial and freshwater ecoregions as modified by The Nature Conservancy was used to define the ecoregions included in this study (http://maps.tnc.org/gis_data.html#ERA; Olson et al., 2001; Abell et al., 2008). Terrestrial ecoregions, as based on those of the World Wildlife Fund (outside the US) and loosely based on Bailey’s ecoregions (from the USDA Forest Service) (within the US), including 867 distinct units within 14 biomes (http://www.worldwildlife.org/biomes; Olson et al., 2001), were utilised as the data for the global terrestrial ecoregions. Freshwater ecoregions followed those proposed by Abell et al. (2008) including 426 distinct units in 12 biomes around the world (http://www.feow.org/).

### Species data

We obtained a list of IPS from the IUCN/SSC Invasive Species Specialist Group (ISSG) including a comprehensive dataset of IPS (http://www.issg.org/) and occurrence data, especially geographic coordinates from the Global Biodiversity Information Facility (GBIF; www.gbif.org; accessed in January 2015). The 387 selected IPS share characteristics including the significant impacts of the IPS on the ecoregion, general functional traits indicating representative issues, and invasion at large scales (e.g. country level) based on the ISSG database (http://www.issg.org/). In total, approximately five million occurrence records of these 387 IPS were collected from GBIF. We used Google Maps to remove occurrence records with the spatial sampling bias according to the following aspects: (1) duplicated records within the area of 10.0-arc-minute spatial resolution; (2) records with both longitude and latitude = 0°; (3) records with equal geographic coordinates (i.e. longitude = latitude); and (4) the records with incorrect species names (https://www.google.com/maps/; http://www.issg.org/; Beck et al., 2014; Aiello-Lammens et al., 2015; García-Roselló et al., 2015). We used 387 species with over 100 records in 10.0 arc-minute pixel cells (16 km at the equator; Araújo et al., 2011) as the input for the climatic suitability model, and 741,114 occurrence records with geographic coordinates were obtained for 387 IPS. We considered the entire globe as the extent of the input data (Table S1; Wisz et al., 2008; Merow, Smith & Silander, 2013; Zhang & Zhang, 2014). We classified the 387 species into nine clusters based on growth forms, such as palm, succulent, alga, fern, aquatic plant, vine, shrub, tree, and herb, according to ISSG (Xu et al., 2018; http://www.issg.org/).

### Bioclimatic data

We used 10.0 arc-minute current and future datasets for the environmental layer input of the species distribution model (Araújo et al., 2011). We obtained nine bioclimatic variables with 10.0-arc-minute spatial resolution (the same as future bioclimatic variables) from the WorldClim database (averages from 1950 to 2000 were used as current bioclimatic variables; www.worldclim.org; Hijmans et al., 2005). The nine bioclimatic variables are shown in Table 1. Hijmans et al. (2005) presented detailed information for bioclimatic variables. The nine bioclimatic variables were selected because they are related to distributions of IPS at global scales and can indicate the maximum, minimum, mean, and variance of temperature and precipitation (Thuiller et al., 2005; Petitpierre et al., 2012; Bellard et al., 2013, 2014). We tested multi-collinearity for the layers of above-mentioned bioclimatic variables using Pearson’s correlation coefficient (*r* ≤ ±0.85) for further analysis (Briscoe et al., 2016). We relied on data from the Intergovernmental Panel on Climate Change Fifth Assessment Report as a reference for modelling the changing trends of IPS invasions (Stocker, 2014; http://www.ipcc.ch/). To model the future potential distribution of IPS in the 2080s (2071–2099), we used the maps of four global climate models (GCMs; i.e. bcc_csm1_1, csiro_mk3_6_0, gfdl_cm3, and mohc_hadgem2_es downloaded from http://www.ccafs-climate.org/), which successfully reproduce the general features of temperature structure in terms of vertical, annual, and inter-annual variation (Thuiller et al., 2005; Bellard et al., 2013, 2014; Kishore et al., 2016). We averaged the pixel values of bioclimatic data based on these four GCMs (Läderach et al., 2017). Representative concentration pathways (RCPs) 4.5 and 8.5 were used in our study (Rogelj, Meinshausen & Knutti, 2012).

**Table 1:** Bioclimatic variables used.

Code	Environmental variables	Unit
Bio1	Annual mean temperature	°C
Bio2	Mean diurnal range	°C
Bio4	Temperature seasonality	SD × 100
Bio5	Maximum temperature of the warmest month	°C
Bio6	Minimum temperature of the coldest month	°C
Bio12	Annual precipitation	mm
Bio13	Precipitation of the wettest month	mm
Bio14	Precipitation of the driest month	mm
Bio15	Precipitation seasonality	C of V

**Note:**

Bioclimatic variables were used as environmental layers for modelling the habitat suitability of IPS by Maxent; C of V represents coefficient of variation.

### Modelling climatic suitability of IPS

Maxent (version 3.3.3k; http://biodiversityinformatics.amnh.org/open_source/maxent/) was used to model the current and future global climatic suitability of the 387 IPS based on current and predicted future bioclimatic data (Phillips, Anderson & Schapire, 2006; Merow, Smith & Silander, 2013). Maxent has the two following characteristics: (1) Maxent has good modelling performance using a small size of occurrence data and (2) Maxent can run using presence points only (Phillips, Anderson & Schapire, 2006; Merow, Smith & Silander, 2013). Many of the worst IPS are still in the process of expanding and can shift their climatic niche due to strong adaptation abilities (Atwater, Ervine & Barney, 2018; Bellard et al., 2018). Consequently, there may be some modelling uncertainties on the prediction of IPS expansion. Although limitations may exist in the climatic suitability modelling approach due to climatic niche shifts, it is necessary to model climatic suitability of IPS under climate change. With the increasing trends of climatic suitability in the target ecoregion, IPS has a greater potential to expand into novel ecoregions (Thuiller et al., 2005; Wiens et al., 2010; Petitpierre et al., 2012). The pixels with an index value greater than zero were identified as the habitats that had the potential to be subjected to plant expansion under climate change.

To precisely predict climatic suitability of IPS, we improved the Maxent modelling performance by optimising the analysis settings based on the study by Merow, Smith & Silander (2013). Specifically, we used the logistic output from Maxent to quantify climatic suitability of IPS under climate change, and we set the regularisation multiplier (beta) to 1.5 to produce a smooth and general response that could be modelled in a biologically realistic manner (Convertino et al., 2014). Then, we used a 10-fold cross-validation approach with 90% of the occurrence data used as a training set, and the remaining 10% of occurrence data was used as the test set in each run of 10 replicates to remove bias with respect to recorded occurrence points (Merow, Smith & Silander, 2013). The modelling output was the average values of 10 replicates in a fold cross-validation approach (Elith et al., 2011; Merow, Smith & Silander, 2013). Hinge features were used for each variable to make linear and threshold features redundant, forming a model with relatively smooth fitted functions (Elith et al., 2011). We set the maximum number of background points as 10,000 to produce pseudo-absences for each IPS, and as much as possible, these background points were close to geographic (and thus environmental) space containing samples of occurrence data to reduce the sampling bias on modelling performance (Phillips et al., 2009). We obtained background points highly correlated with true probability of presence using the presence–absence modelling purposed by Phillips et al. (2009). The other settings were the same as described in Elith et al. (2011) and Phillips, Anderson & Schapire (2006).

We evaluated the predictive precision of Maxent using the area under the curve (AUC) of the receiver operation characteristic, which regards each value of the prediction result as a possible threshold, before obtaining the corresponding sensitivity and specificity through calculations (Raes & Ter Steege, 2007). However, using AUC only is not enough to assess the predictive precision of Maxent (Lobo, Jiménez-Valverde & Real, 2008; Leroy et al., 2018). Here, we used the average omission rates of training occurrence records to further assess the predictive precision of Maxent for each IPS according to six thresholds of distribution presence. These thresholds included fixed cumulative value five, fixed cumulative value 10, equal training sensitivity and specificity, maximum training sensitivity plus specificity, maximum test sensitivity plus specificity, and equate entropy of threshold and original distributions (Phillips, Anderson & Schapire, 2006; Merow, Smith & Silander, 2013). When AUC values were above 0.7, and meanwhile the average omission rates of training occurrence records were less than 0.017, the modellings were included in our study (Phillips, Anderson & Schapire, 2006; Elith et al., 2011; Hijmans, 2012; Merow, Smith & Silander, 2013). *Poa pratensis* with AUC values less than 0.7 was not considered in our downstream analyses. The other 386 species were included in our analysis (detailed information in Table S1). The 386 IPS were also widely distributed over the Earth based on our occurrence records.

### Potential of invasive plant expansion

Previous studies (Thuiller et al., 2005; Bellard et al., 2013) used a fixed threshold to match invasive plant expansion at pixel levels from the results of climatic suitability modellings. However, some studies (Calabrese et al., 2014; Merow, Smith & Silander, 2013) have indicated that thresholds are problematic and can produce bias in predictions for multi-species distribution patterns. Here, we used a likelihood approach (Calabrese et al., 2014) to assess expansion potential of multi-IPS in each pixel.

First, we used the modified method described by Calabrese et al. (2014) to compute the climatic suitability of multi-IPS in each pixel:

$${E_j} = \sum\limits_{i = k}^k {{P_{i,j}}} $$

where *E_j_* represents the current or future climatic suitability of multi-IPS in pixel *j*; *k* is the number of IPS in pixel *j*; and *P_i_,_j_* is the climatic suitability of multi-IPS in pixel *j*.

We calculated the change of climatic suitability of multi-IPS between current conditions and the 2080s (RCPs 4.5 and 8.5) in each pixel:

$${A_j} = E_j^f-E_j^c$$

where *A_j_* is the change of climatic suitability of multi-IPS between current conditions and the 2080s (i.e. RCPs 4.5 and 8.5) in pixel *j*, and *E*_*j*_^*c*^ and *E*_*j*_^*f*^ are the climatic suitability of multi-IPS in pixel *j* in current and future (i.e. RCPs 4.5 and 8.5), respectively. Hence, the pixels with a large difference of climatic suitability indicate a high suitability for multi-IPS between current and future climate scenarios. Moreover, a small but positive trend of multi-IPS climatic suitability can represent a potential range expansion of IPS in specific ecoregions.

Then, we summed the change values of climatic suitability of pixels for multi-IPS between current conditions and the 2080s (RCPs 4.5 and 8.5) to quantify the potential of IPS expansion in each ecoregion. In our study, the ecoregions with changes in multi-IPS climatic suitability between the 2080s and current conditions over 0 were included, and the ones with changes less than 0 were excluded. We summed the values of the possibilities for IPS expansion in ecoregions based on the biomes and growth forms. We used a linear regression analysis to assess the relationship between the potentials for IPS expansion in RCPs 4.5 and 8.5 based on each ecoregion biome and growth form. We found that there was a significant relationship between the potentials of IPS expansion in RCPs 4.5 and 8.5 (*P* < 0.001; Table S2). Therefore, RCP 4.5 was used to show our results.

### Climatic features of ecoregion analysis with high potential of invasive plant expansion

We determined the most important variables for climatic suitability of IPS using the Jackknife test in Maxent (Papeş & Gaubert, 2007; Phillips & Dudík, 2008; Merow, Smith & Silander, 2013). Then, we extracted the average value of the climatic variable from the Jackknife test, which is the most important to the climatic suitability of IPS in each ecoregion. We used the following equation to compute the changes of important climate variables in each ecoregion:

$${\rm{C}}{{\rm{V}}_n} = V_n^f-V_n^c$$

where CV_*n*_ is the change of important climate variables in the ecoregion *n*; *V*_*j*_^*f*^ and *V*_*n*_^*c*^ are the future and current climate variables in ecoregion *n*, respectively.

A linear regression analysis was also used to compute the relationship between the IPS potential to expand in ecoregions and the change of important climate variables. This was based on the biomes for exploring the climatic features of ecoregions with a high potential of IPS expansion under climate change (Peterson et al., 2008). Finally, we calculated the mean and standard deviation for the changes of important climate variables between current and future scenarios (i.e. RCP 4.5) based on different biomes.

## Results

### Invasive plant expansion potential in ecoregions

**Figure 1:**
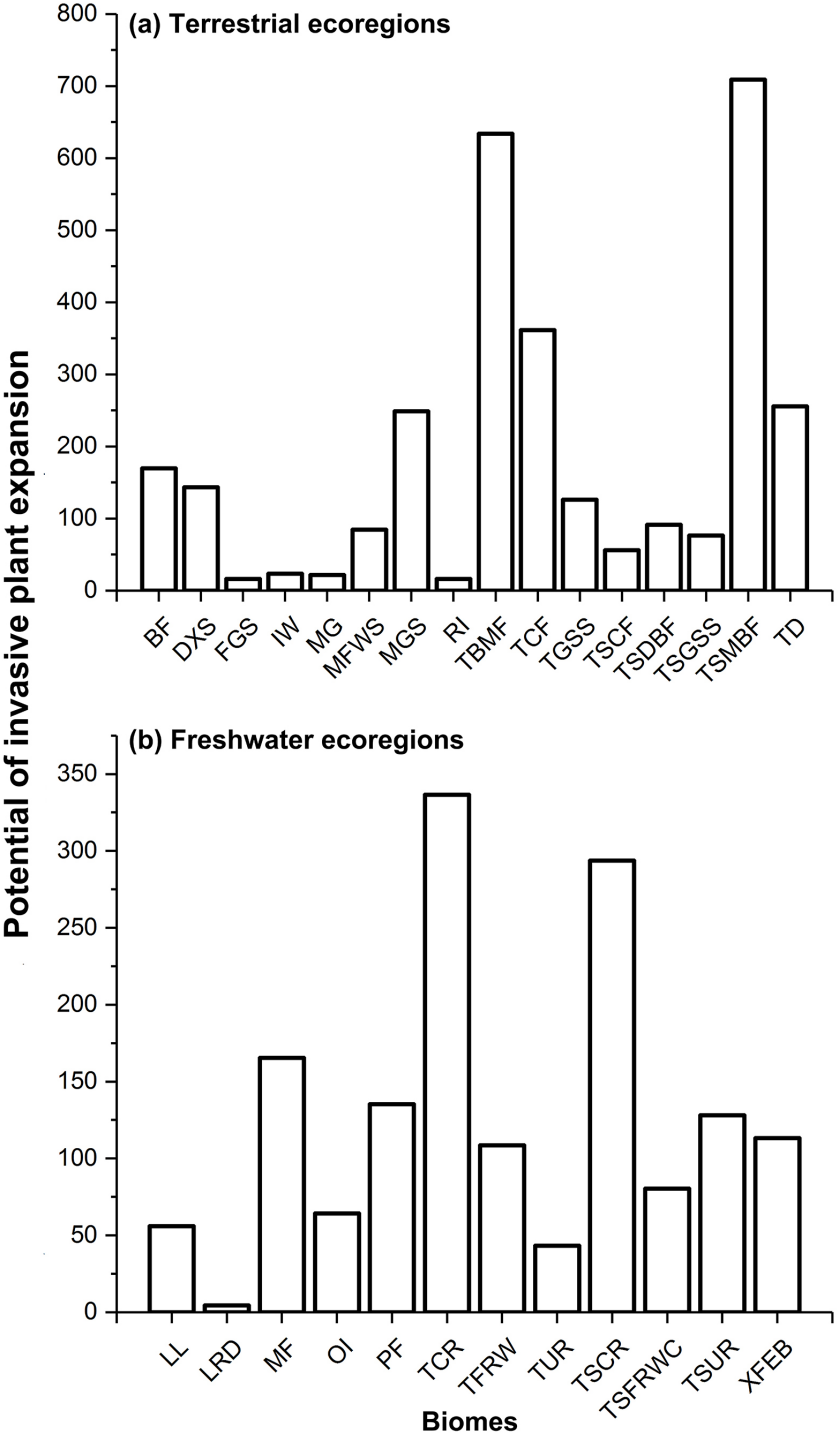
Potential of invasive plant expansion in terrestrial (A) and freshwater (B) ecoregions in RCP 4.5. The numbers of this figure represent the degrees of invasive plant expansion potential based on the sum values on change of climatic suitability of pixels for multi-IPS between current conditions and the 2080s (RCPs 4.5 and 8.5) at ecoregional levels. Terrestrial represents terrestrial ecoregions; Freshwater represents freshwater ecoregions; Codes used in this figure are defined as follows: For terrestrial ecoregions: BF, boreal forests/taiga; DXS, deserts and xeric shrublands; FGS, flooded grasslands and savannas; IW, inland water; MG, mangroves; MFWS, Mediterranean forests, woodlands and scrub; MGS, montane grasslands and shrublands; RI, rock and ice; TBMF, temperate broadleaf and mixed forests; TCF, temperate conifer forests; TGSS, temperate grasslands, savannas and shrublands; TSCF, tropical and subtropical coniferous forests; TSDBF, tropical and subtropical dry broadleaf forests; TSGSS, tropical and subtropical grasslands, savannas and shrublands; TSMBF, tropical and subtropical moist broadleaf forests; TD, tundra. For freshwater ecoregions: LL, large lakes; LRD, large river deltas; MF, montane freshwaters; OI, oceanic islands; PF, polar freshwaters; TCR, temperate coastal rivers; TFRW, temperate floodplain rivers and wetlands; TUR, temperate upland rivers; TSCR, tropical and subtropical coastal rivers; TSFRWC, tropical and subtropical floodplain rivers and wetland complexes; TSUR, tropical and subtropical upland rivers; XFEB, xeric freshwaters and endorheic (closed) basins.

**Figure 2:**
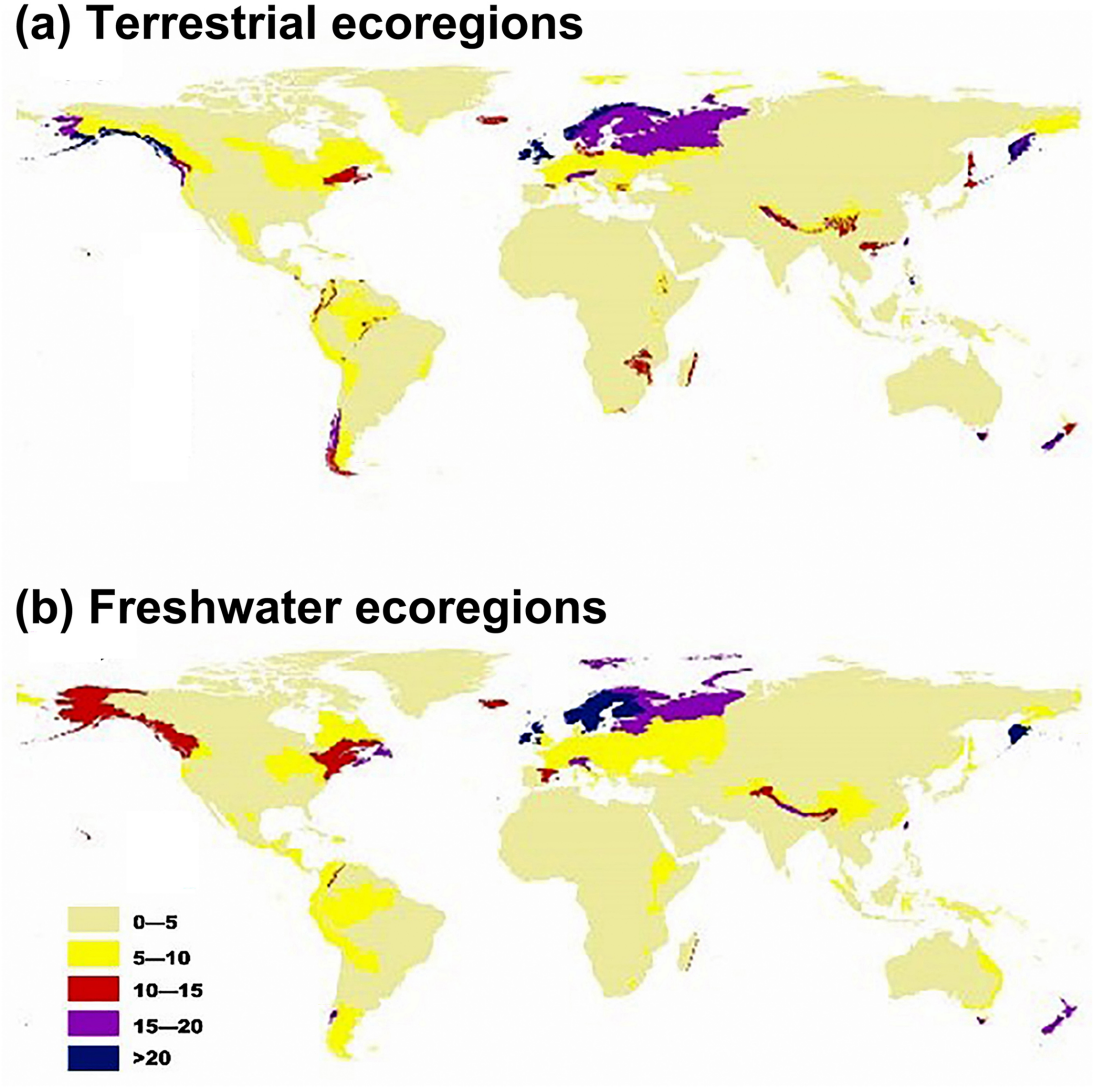
Map showing the potential for invasive plant expansion in RCP 4.5 for terrestrial ecoregions (A) and freshwater ecoregions (B). The colours coupled with the numbers in this figure represent the level of IPS expansion potential across different ecoregions. Blue means there is a very high chance of expansion and tan-yellow means a low chance of expansion. The ecoregion maps were obtained from the studies of Olson et al. (2001) and Abell et al. (2008).

Regarding terrestrial ecoregions, IPS, such as aquatic plants, trees, vines, and herbs had the largest potential to expand in Montane Grasslands and Shrublands, Temperate Broadleaf and Mixed Forests, Temperate Conifer Forests, Tropical and Subtropical Moist Broadleaf Forests, and Tundra (Figs. 1A and 2). For freshwater ecoregions including Tropical and Subtropical Coastal Rivers, Temperate Coastal Rivers, Xeric Freshwaters, Endorheic (closed) Basins, and Montane Freshwaters, the biomes would be severely impacted by the expansion of IPS (Figs. 1B and 2). These ecoregions are mainly distributed in Northern Europe, the UK, South America, North America, southwest China, and New Zealand (Fig. 2).

### Climatic features of ecoregions with high invasive plant expansion potential

**Figure 3:**
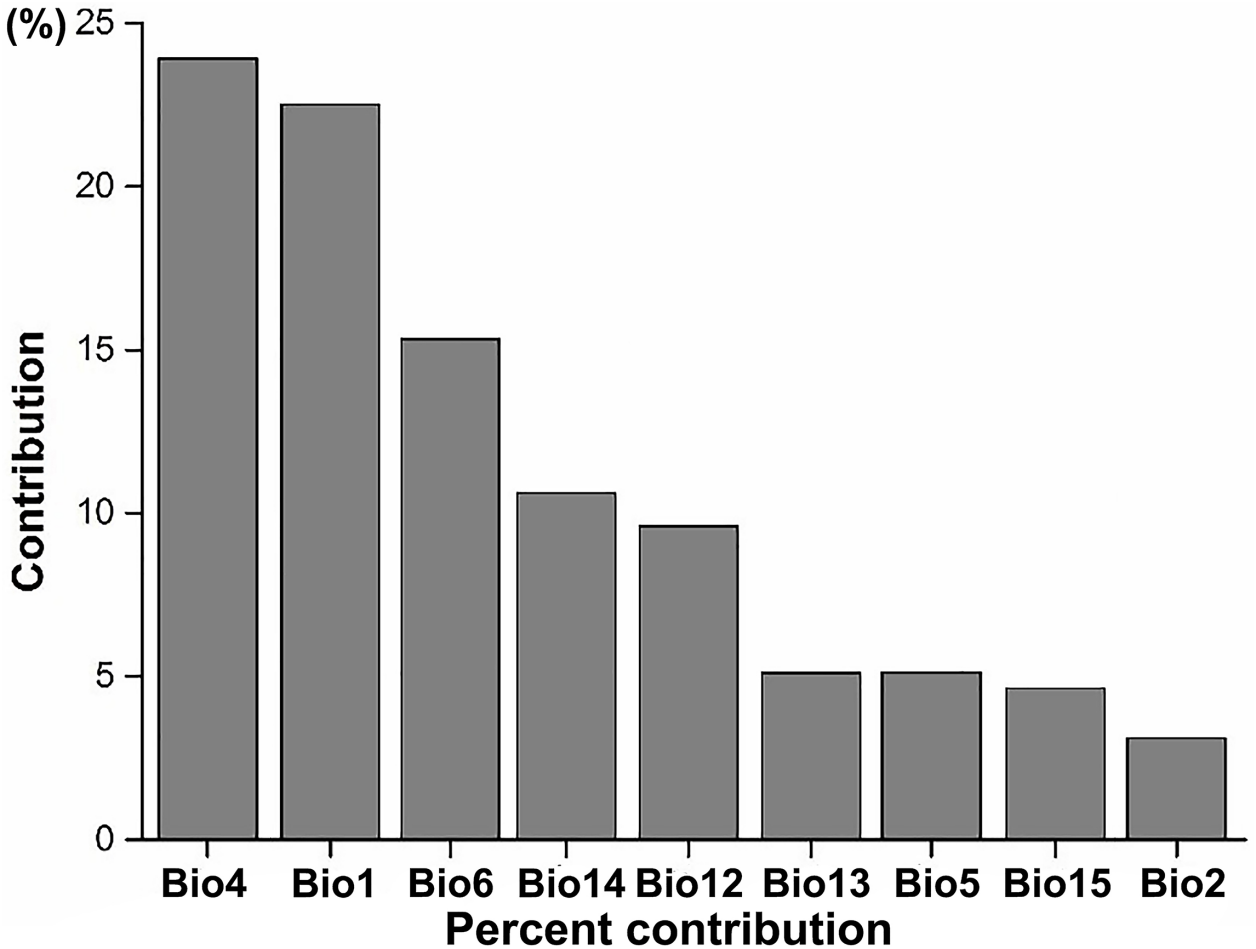
The average percent contribution of climatic variables to climatic suitability of IPS based on a Jackknife test in Maxent. Bio1, annual mean temperature (°C); Bio2, mean diurnal range (°C); Bio4, temperature seasonality; Bio5, maximum temperature of the warmest month (°C); Bio6, minimum temperature of the coldest month (°C); Bio12, annual precipitation (mm); Bio13, precipitation in the wettest month (mm); Bio14, precipitation in the driest month (mm); Bio15, precipitation seasonality (mm).

According to the results of the Jackknife test, we found that the most important climatic suitability variables for IPS were annual mean temperature and temperature seasonality (Fig. 3; Table S1), indicating that there was a significant linear relationship between the changes of annual mean temperature between current and RCP 4.5 scenarios and potential of IPS to expand in ecoregions across different biomes (*P* < 0.05). The biomes most affected include the following: terrestrial ecoregions—Rock and Ice, Temperate Broadleaf and Mixed Forests, Temperate Grasslands, Savannas and Shrublands, and Tropical and Subtropical Coniferous Forests (Table 2); freshwater ecoregions—Xeric Freshwaters and Endorheic (closed) Basins (Table 2). For temperature seasonality, we also found a similar linear relationship to annual mean temperature. The biomes most affected include the following: terrestrial ecoregions—Montane Grasslands and Shrublands, Temperate Broadleaf and Mixed Forests, Temperate Conifer Forests, and Tropical and Subtropical Moist Broadleaf Forests (Table 2); freshwater ecoregions—Large Lakes, Tropical and Subtropical Upland Rivers, and Tropical and Subtropical Floodplain Rivers and Wetland Complexes (Table 2).

**Table 2:** The determination coefficients (*R*^2^) for relationships between climatic variables and the invasive plant expansion potential in ecoregions.

Code	RCP 4.5-*R*^2^		RCP 8.5-*R*^2^	
	Bio1	Bio4	Bio1	Bio4
BF	0.0044^ns^	0.0066^ns^	0.0258^ns^	0.0051^ns^
DXS	0.0056^ns^	0.0142^ns^	0.0022^ns^	0.0695*
FGS	0.0155^ns^	0.1448^ns^	0.1051^ns^	0.3650**
IW	0.0891^ns^	0.5629^ns^	0.3691^ns^	0.4882^ns^
MG	0.0004^ns^	0.0001^ns^	0.0490^ns^	0.0012^ns^
MFWS	0.0046^ns^	0.0037^ns^	0.0043^ns^	0.0030^ns^
MGS	0.0084^ns^	0.1486**	0.0094^ns^	0.2255***
RI	0.9939*	0.9904^ns^	0.9967*	0.9376^ns^
TBMF	0.2116***	0.0503*	0.3128***	0.1067***
TCF	0.0151^ns^	0.2061**	0.0205^ns^	0.2395***
TGSS	0.4035***	0.0018^ns^	0.4271***	0.0070^ns^
TSCF	0.2494*	0.0026^ns^	0.1143^ns^	0.0118^ns^
TSDBF	0.0000^ns^	0.0150^ns^	0.0116^ns^	0.0377^ns^
TSGSS	0.0331^ns^	0.0019^ns^	0.0364^ns^	0.0045^ns^
TSMBF	0.0113^ns^	0.0261*	0.0012^ns^	0.0027^ns^
TD	0.0007^ns^	0.0124^ns^	0.0109^ns^	0.0757^ns^
LL	0.1231^ns^	0.3633*	0.1642^ns^	0.3118*
LRD	0.0427^ns^	0.1688^ns^	0.0107^ns^	0.2217^ns^
MF	0.0058^ns^	0.0003^ns^	0.0079^ns^	0.0475^ns^
OI	0.0533^ns^	0.0245^ns^	0.0903^ns^	0.0332^ns^
PF	0.1700^ns^	0.0978^ns^	0.1427^ns^	0.1040^ns^
TCR	0.0182^ns^	0.0292^ns^	0.0038^ns^	0.0597^ns^
TFRW	0.0872^ns^	0.0875^ns^	0.0066^ns^	0.0406^ns^
TUR	0.0012^ns^	0.0388^ns^	0.0938^ns^	0.0233^ns^
TSCR	0.0267^ns^	0.0387^ns^	0.0022^ns^	0.0009^ns^
TSFRWC	0.0011^ns^	0.1550**	0.1976*	0.0395^ns^
TSUR	0.0314^ns^	0.3912***	0.0140^ns^	0.5492***
XFEB	0.0742*	0.0429^ns^	0.0275^ns^	0.0910*

**Notes:**

Bio1 represents annual mean temperature; Bio4 represents temperature seasonality. Abbreviations used in this figure are defined as follows: BF, boreal forests/taiga; DXS, deserts and xeric shrublands; FGS, flooded grasslands and savannas; IW, inland water; MG, mangroves; MFWS, Mediterranean forests, woodlands and scrub; MGS, montane grasslands and shrublands; RI, rock and ice; TBMF, temperate broadleaf and mixed forests; TCF, temperate conifer forests; TGSS, temperate grasslands, savannas and shrublands; TSCF, tropical and subtropical coniferous forests; TSDBF, tropical and subtropical dry broadleaf forests; TSGSS, tropical and subtropical grasslands, savannas and shrublands; TSMBF, tropical and subtropical moist broadleaf forests; TD, tundra. For freshwater ecoregions: LL, large lakes; LRD, large river deltas; MF, montane freshwaters; OI, oceanic islands; PF, polar freshwaters; TCR, temperate coastal rivers; TFRW, temperate floodplain rivers and wetlands; TUR, temperate upland rivers; TSCR, tropical and subtropical coastal rivers; TSFRWC, tropical and subtropical floodplain rivers and wetland complexes; TSUR, tropical and subtropical upland rivers; XFEB, xeric freshwaters and endorheic (closed) basins.

* *P* < 0.05*.

** *P* < 0.01.

*** *P* < 0.001.

ns *P* > 0.05.

**Figure 4:**
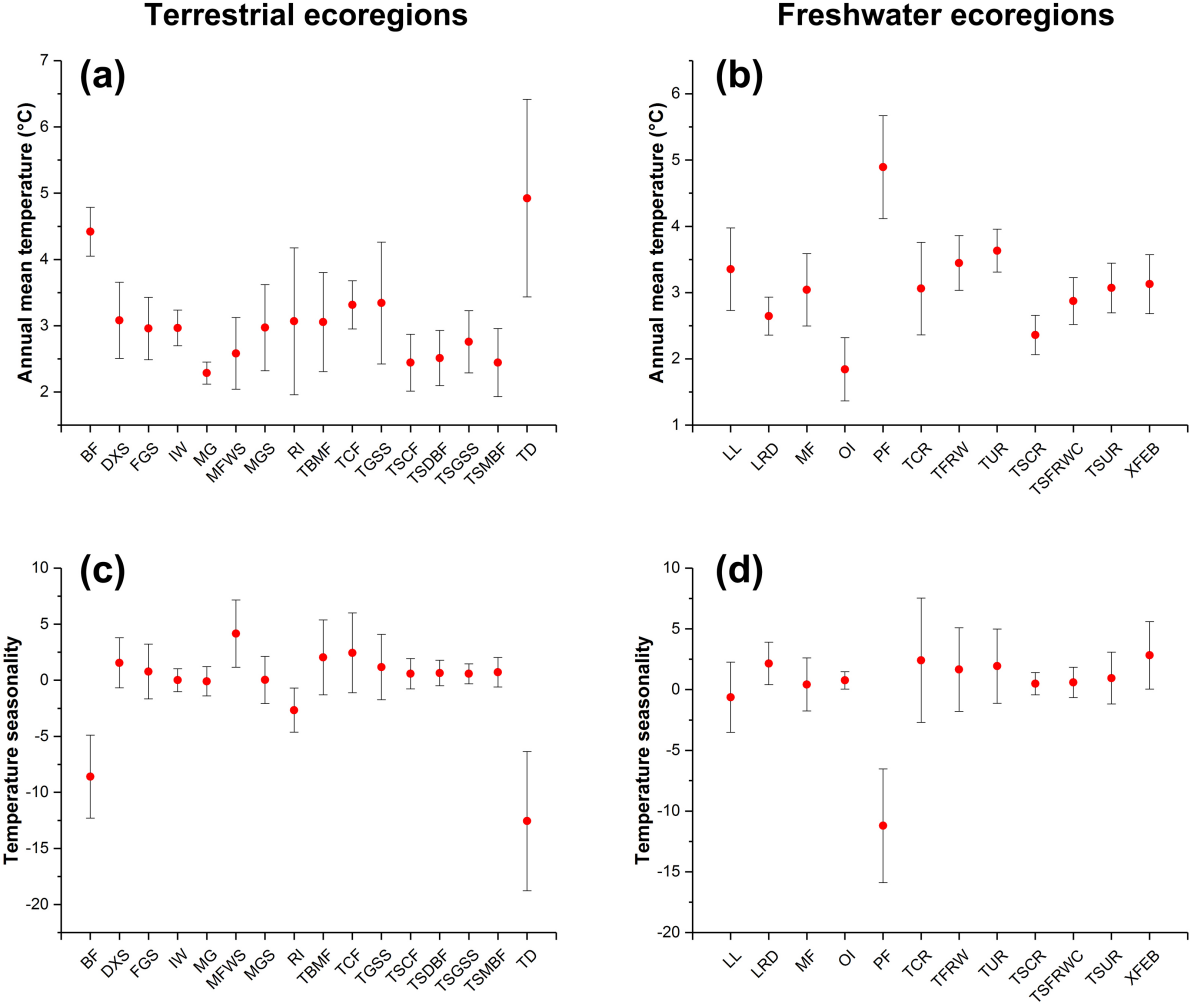
The changes in annual mean temperature (A and B) and temperature seasonality (C and D) of ecoregions with expansion potential of IPS across different biomes between current and RCP 4.5 scenarios. The red points represent the average changes in annual mean temperature and temperature seasonality of ecoregions with expansion potential of IPS for each biome. The bars represent the standard deviation of changes in annual mean temperature and temperature seasonality of ecoregions with expansion potential of IPS for each biome.

We found that climatic features of terrestrial ecoregions with a high IPS expansion potential (i.e. Montane Grasslands and Shrublands, Temperate Broadleaf and Mixed Forests, Temperate Conifer Forests, Tropical and Subtropical Moist Broadleaf Forests, and Tundra) had relatively large changes in annual mean temperature and temperature seasonality between current and RCP 4.5 scenarios (Fig. 4). The freshwater ecoregions of high expansion potential (i.e. Tropical and Subtropical Coastal Rivers, Temperate Coastal Rivers, Xeric Freshwaters and Endorheic (closed) Basins, and Montane Freshwaters) may be distributed in ranges with large changes in temperature seasonality between current and RCP 4.5 scenarios. However, climatic features of freshwater ecoregions with a high expansion potential differ depending on a variety of biomes (Fig. 4).

## Discussion

### Invasive plant expansion potential in global ecoregions under climate change

This study evaluated and mapped the potential expansion of IPS in global ecoregions due to climate change. Climate change could increase the potential expansion of IPS, including aquatic plants, trees, and herbs to spread in the ecoregions distributing in Northern Europe, the UK, South America, North America, southwest China, and New Zealand. We found that climate change would drive IPS into coastal biomes or high latitude areas and suppress the growth of alpine, temperate, and coastal plants. Steinbauer et al. (2018) have shown that the likelihood for plant species richness to increase on mountain summits is linked to climate warming, indicating that acceleration in climate-induced biotic change is occurring even in remote places on Earth, with potentially far-ranging consequences for both biodiversity and ecosystem functions. Previous studies coupled with our results have shown that climate change would increase the risk of IPS in coastal regions or high latitude areas (Peterson et al., 2008; Chen et al., 2011; Petitpierre et al., 2016); however, Tundra is an exception. Tundra is a biome in which low temperatures and short growing seasons hinder tree growth (Olson et al., 2001). IPS fail to become established in Tundra biomes due to limited resource fluctuation, low productivity, and low human disturbance (Olson et al., 2001; Kalusová et al., 2013).

Invasion of IPS has a large potential to result in landscape homogeneity at ecoregional scales. IPS can compete with native plant species and occupy available habitats and resources in invaded ranges at large scales (Callaway & Aschehoug, 2000; Vila & Weiner, 2004; Price, Spyreas & Matthews, 2018). Hence, the species richness of native plants would be threatened by IPS expansion. This wide geographical distribution and limited taxonomic diversity of native plants creates greater inherent taxonomic homogeneity due to IPS expansion (Hellmann et al., 2008; Ekroos, Heliölä & Kuussaari, 2010; Price, Spyreas & Matthews, 2018). IPS expansion can make the prospect of homogenisation and loss of biodiversity a substantial conservation concern due to climate change (Ekroos, Heliölä & Kuussaari, 2010; Price, Spyreas & Matthews, 2018). Furthermore, numerous ecoregions are vulnerable and endangered due to biological invasion (Olson & Dinerstein, 1998). Our results have shown that climate change could promote IPS to expand in ecoregions of Northern Europe, the UK, South America, North America, southwest China, and New Zealand, indicating that IPS expansion could lead to homogenisation and biodiversity loss in ecoregions.

Specifically, climate change could affect the ecologically suitable areas for invasive trees and herbs, helping affected species persist against local enemies (Didham et al., 2007; Maron et al., 2014). Furthermore, IPS with niche conservatism would invade habitats similar to their native range (Petitpierre et al., 2012). IPS, particularly herbs, also have broad niche breadths (Petitpierre et al., 2012). Large areas of natural habitats could be severely invaded by IPS across different spatial scales (Bradley, Oppenheimer & Wilcove, 2009; Bradley, Wilcove & Oppenheimer, 2010; Petitpierre et al., 2016). These specific invasion characteristics could cause invasive trees and herbs to impact on native plants, limiting the suitable habitat availability for native species and even leading to biodiversity loss (Didham et al., 2007; Funk & Vitousek, 2007). Moreover, further development of trade networks, human travel, and environmental change would promote the invasion of aquatic plants (Rahel & Olden, 2008; Donaldson et al., 2014; Gillard et al., 2017). Thus, invasive aquatic plants could negatively affect the water quality and constrict the available habitats of native species (Crooks, 2002; Donaldson et al., 2014; Bajer et al., 2016; Gillard et al., 2017).

### The role of climate factors on invasive plant expansion potential

Our findings suggest that the variable most important to climatic suitability for IPS was annual mean temperature and temperature seasonality indicated that temperature could affect the IPS expansion potential in the ecoregions. The ecoregions with large changes of annual mean temperature and temperature seasonality would be severely invaded by IPS in Montane Grasslands and Shrublands, Temperate Forests, Tundra, and some Tropical and Subtropical Moist Broadleaf Forests. For freshwater ecoregions, IPS also had the potential to expand in the regions with large changes in temperature seasonality. These freshwater regions included Coastal and Polar regions. However, Tundra and Polar regions are extremely unsuitable for IPS in current climate conditions (Kalusová et al., 2013). Hence, we need to attach importance to IPS expansion into coastal or high latitude ecological systems, such as temperate forests, alpine habitats, and coastal rivers, under climate change.

In addition, we also found that there was a significant linear relationship between temperature changes and the potential of IPS to expand in biomes, indicating that the potential of IPS to invade ecoregions could be predicted by reasonable monitoring (Bradley, Wilcove & Oppenheimer, 2010; Early et al., 2016). Some studies have shown extreme climatic events, such as unusual heat waves, hurricanes, floods, and droughts; facilitating invasions of IPS (White et al., 2001; Diez et al., 2012). Although our data suggests that IPS could not severely invade the ecoregions listed above, we need to prevent the escalated risk of IPS by extreme climatic events in these ecoregions (Diez et al., 2012). Moreover, these linear relationships provided insight into ecological restoration (Bradley, Oppenheimer & Wilcove, 2009). When we take action to restore ecoregions, such as Temperate Broadleaf and Mixed Forests, Temperate Grasslands, Savannas and Shrublands, Large Lakes, and Tropical and Subtropical Floodplain Rivers and Wetland Complexes, we should consider the role of climate factors on the potential for IPS invasion during ecological restoration.

We should pay attention to some southern areas like New Zealand and South Africa, where native plants have little competition strength (Dzikiti et al., 2013; Suckling, 2013; Ellender & Weyl, 2014; Nuñez & Dickie, 2014). The isolation of some regions has an effect on species invasion potential as a response to the historical patterns of plant distribution. Previous studies (Gimeno, Vila & Hulme, 2006; Sheppard, 2013) have shown that hat local processes (i.e. the biotic resistance of plant communities) are less important than large-scale phenomena (i.e. environmental driving forces), and climate factors are the main forces of plant invasion in New Zealand and South Africa (Gimeno, Vila & Hulme, 2006; Sheppard, 2013; Donaldson et al., 2014). Furthermore, weak competition ability of native plant species may enhance the role of climate factors on invasive plant expansion potential in southern areas (e.g. New Zealand and South Africa; Callaway & Aschehoug, 2000; Vila & Weiner, 2004; Dzikiti et al., 2013; Suckling, 2013; Ellender & Weyl, 2014). Therefore, climate factors play an important role on invasive plant expansion potential of ecoregions at global scales.

### Implication for invasion management

Based on our results, we provide suggestions for invasion management under climate change. Monitoring strategies should be defined and utilised for climate change for IPS, particularly for aquatic plants, trees, and herbs in the biomes of coastal regions or high latitudes (Petitpierre et al., 2016; Wang, Wan & Zhang, 2017). In these ecoregions, climate change could result in a number of potential consequences for IPS in areas with a high invasion potential, such as changing transport and introduction mechanisms, establishment of new IPS in invaded regions, impact of existing IPS on invaded habitats, redistribution of existing IPS, and reduction in effectiveness of control strategies (Thuiller et al., 2005; Hellmann et al., 2008; Cronin et al., 2014; Early et al., 2016). Early et al. (2016) have shown that areas with high levels of poverty and low historical levels of invasion may be severely invaded by IPS. These consequences would result in a large potential for IPS to impact regional ecoregions worldwide. Hence, we need to design long-term management plans at the biome scale to create a mitigation strategy for the expansion of IPS in ecoregions due to climate change (Olson et al., 2001; Abell et al., 2008; Early et al., 2016). We should also develop policies to prevent intentional or accidental introduction or IPS dispersal worldwide (Powell, Chase & Knight, 2011; Kalusová et al., 2013). Considering forest and coastal biomes, we need to create a framework of adaptive management for forest and aquatic IPS under climate change (Kulhanek, Ricciardi & Leung, 2011; Donaldson et al., 2014; Bajer et al., 2016; Gillard et al., 2017). Furthermore, Early et al. (2016) have shown that plant invasion may be a current result of environmental change in economically developing regions. Hence, combined with our results, we need to attach importance to the improvement of early-warning monitoring schemes in the ecoregions with coastal or high latitudes in developing countries (Early et al., 2016; Petitpierre et al., 2016).

Furthermore, our results showed that large changes in temperature seasonality between current and future scenarios may lead to a high potential for IPS to expand in ecoregions (e.g. Montane Grasslands and Shrublands, Temperate Broadleaf and Mixed Forests, Temperate Conifer Forests, Tropical and Subtropical Coastal Rivers, and Temperate Coastal Rivers), indicating that we should include temperature seasonality features of ecoregions with high expansion potentials into early-warning monitoring schemes for invasion management. We also need to pay attention to the changes in annual mean temperature within ecoregions. Our results showed that the increasing annual mean temperature may result in a high expansion of IPS in terrestrial ecoregions at a large scale, but the effects of annual mean temperature on plant invasion may depend on the type of biome for freshwater ecoregions. Hence, we could propose detailed references on prevention and control of IPS expansion at a large scale and delineate the regions with a high risk of plant invasion around the world (Bradley, Wilcove & Oppenheimer, 2010; Van Kleunen et al., 2015; Fig. 4).

Finally, we need to determine the exchange pathways of IPS in ecoregions around the world and establish a monitoring network of geographic information for IPS expansion in ecoregions. Previous research has presented a comprehensive analysis of global accumulation and exchange pathways of IPS across continents and provided important references for the prevention of IPS expansion by human-mediated dispersal of species into new regions (Van Kleunen et al., 2015). Furthermore, climatic suitability coupled with human activities explains most of the variation in establishment for IPS across different continents (Kalusová et al., 2013; Donaldson et al., 2014; Feng et al., 2016). Combined with our assessment of the expansion potential of IPS across global ecoregions, we should integrate exchange pathways of IPS across native and invaded ranges into a global monitoring network for invasion risk under climate change. For example, *A. mearnsii*, native to Australia, could invade South Africa and cause ecological, economic, and social damage in invaded ranges (Le Maitre et al., 2002). Donaldson et al. (2014) proposed an approach to manage the invasion risk of *A. mearnsii* in South Africa by identifying the exchange pathways between Australia and South Africa. Hence, such determination of exchange pathways could be based on ecoregion scales due to climate change.

## Limitations

Although our study provided an evaluation of the global expansion of IPS, the following limitations remain.

First, we took both invasive and native ranges into consideration for the global assessment of the spread of IPS. The native and invasive ranges were not separated, thus there may be bias for our results (Essl et al., 2018). However, the IPS that we selected could result in potentially serious ecosystem and biodiversity harm (http://www.issg.org/). Furthermore, the ecoregional boundary of invasive ranges (i.e. obvious geographic distribution barrier) could not be definitively identified (Essl et al., 2018). Hence, the consideration of extensive ranges at global scales is necessary for invasion assessment for IPS.

Second, AUC, which is a presence-absence metric, may not be a good measure of model robustness in presence-background (Lobo, Jiménez-Valverde & Real, 2008; Leroy et al., 2018). Hence, we used a plausible alternative (i.e. the omission rates of training occurrence records) to assess the predictive precision of Maxent coupled with AUC (Phillips, Anderson & Schapire, 2006; Merow, Smith & Silander, 2013). Future studies should use occurrence records of fieldwork to assess the accuracy of Maxent modelling.

Third, we made an assumption in the methods, stating that plant species will have stable climatic suitability in their native and invasive regions (Petitpierre et al., 2012). Such an assumption is still debatable (Petitpierre et al., 2012; Stigall, 2014; Atwater, Ervine & Barney, 2018). It is also unknown whether Maxent modelling could capture the entire IPS niche. In our study, we integrated the occurrence records of native, non-native, and invasive ranges into our modelling to include more niches (Donaldson et al., 2014). Hence, we could reduce the uncertainties of niche conservatism of IPS between invasive and native ranges. Future studies should use a more extensive database of occurrence records to improve robustness of climatic suitability modellings for plant invasion assessment across global biomes.

Fourth, we did not divide terrestrial and aquatic IPS into terrestrial and freshwater biomes, respectively. It is difficult to define the habitats of IPS because IPS may have both terrestrial and freshwater habitats due to the inherent plasticity of evolution and adaptation of IPS to rapid environmental changes (Hoffmann & Sgrò, 2011; Essl et al., 2018).

Fifth, the likelihood of invasions depends upon many factors, for example, regions of origin, regions of destination, human usage, likelihood of being transported, and sensitivity of invaded regions, which altogether influence the different stages of invasion: introduction, establishment, spread, and impacts (Donaldson et al., 2014; Early et al., 2016; Bellard et al., 2018; Essl et al., 2018). The relevant factors should be considered for future studies.

Sixth, the high AUCs obtained in our study may be due to the background points extracted from areas geographically and ecologically larger than the range of any given species (Acevedo et al., 2012). Here, we used the omission rates to assess the Maxent modelling performance. Future studies could determine the background points of IPS based on the ecoregional ranges due to the similarities across complex multivariate environmental factors by grouping areas into similar categories.

Seventh, previous studies (Breiner et al., 2015; Mainali et al., 2015; Beaumont et al., 2016; Briscoe et al., 2016) have shown that ensemble modellings have better performance for prediction of current and future distributions than a single algorithm (Thuiller et al., 2008). Furthermore, the modelling transferability may be low. However, some modellings (i.e. general linear modelling) need real absence points. Hence, it is still a challenge to assess IPS expansion potential at large scales due to the lack of real absence points. Here, we suggested to determine the real absence points based on the ranges of presence points and ecoregions across different time periods using the method of Phillips et al. (2009).

Eighth, our study could not decrease the uncertainties on the static modelling approach and lack of integration with current modelling approaches at the landscape level. Mechanistic modelling should be developed to reduce modelling prediction uncertainties in the future studies. The understanding and quantification of long-distance seed dispersal have been paid attention in recent years (Thuiller et al., 2008; Feng et al., 2016). Furthermore, generation time is a key factor affecting the evolutionary potential of IPS along rapid climatic change (Dukes & Mooney, 1999; Thuiller et al., 2008). Hence, we should take movement ability and biotic factors (e.g. long-distance seed dispersal and generation time) into consideration for the use of climatic suitability modellings on plant invasion assessment across different biomes (Thuiller et al., 2008; Kalusová et al., 2013; Donaldson et al., 2014; Briscoe et al., 2016; Feng et al., 2016).

Despite these limitations, Maxent is still a robust model for predicting climatic suitability of IPS at large scales based on presence points only, and a likelihood approach (Calabrese et al., 2014) should be used to assess plant invasion across different biomes. Although the abovementioned issues are present in our study, an assessment of global invasion is important at ecoregional levels.

## Conclusion

Our study provided a global method to evaluate the present and future expansion of IPS and is a resource for the prevention and control of IPS. We found that global climate change would cause IPS, such as aquatic plants, trees, and herbs to attack global ecoregions by expanding in coastal ecoregions or high latitudes. Plant invasion has a large potential to be enhanced due to the process of economic globalisation and rapid climate change. Therefore, the risk evaluation of universal coverage for IPS is urgently needed at a global scale.

## Supplemental Information

10.7717/peerj.6479/supp-1 Supplemental Information 1 Table S1. IPS, AUC values, omission rates and the results of Maxent jackknife test.

10.7717/peerj.6479/supp-2 Supplemental Information 2 Table S2. The relationships of the expansion potential of IPS between RCPs 4.5 and 8.5 scenarios based on logistic values of Maxent. Terrestrial represents terrestrial ecoregions; Freshwater represents freshwater ecoregions; Codes used in this table are defined as follows: For terrestrial ecoregions: BF: Boreal Forests/Taiga; DXS: Deserts and Xeric Shrublands; FGS: Flooded Grasslands and Savannas; IW: Inland Water; MG: Mangroves; MFWS: Mediterranean Forests, Woodlands and Scrub; MGS: Montane Grasslands and Shrublands; RI: Rock and Ice; TBMF: Temperate Broadleaf and Mixed Forests; TCF: Temperate Conifer Forests; TGSS: Temperate Grasslands, Savannas and Shrublands; TSCF: Tropical and Subtropical Coniferous Forests; TSDBF: Tropical and Subtropical Dry Broadleaf Forests; TSGSS: Tropical and Subtropical Grasslands, Savannas and Shrublands; TSMBF: Tropical and Subtropical Moist Broadleaf Forests; TD: Tundra. For freshwater ecoregions: LL: Large Lakes; LRD: Large River Deltas; MF: Montane Freshwaters; OI: Oceanic Islands; PF: Polar Freshwaters; TCR: Temperate Coastal Rivers; TFRW: Temperate Floodplain Rivers and Wetlands; TUR: Temperate Upland Rivers; TSCR: Tropical and Subtropical Coastal Rivers; TSFRWC: Tropical and Subtropical Floodplain Rivers and Wetland Complexes; TSUR: Tropical and Subtropical Upland Rivers; XFEB: Xeric Freshwaters and Endorheic (closed) Basins. *P* < 0.05*; *P* < 0.01**; *P* < 0.001***.

10.7717/peerj.6479/supp-3 Supplemental Information 3 Fig. S1. Potential of invasive plant expansion in terrestrial and freshwater ecoregions in RCPs 4.5 and 8.5. The color coupling with the numbers of this figure represents the level of IPS expansion potential across different ecoregions. The numbers of this figure represent the degrees of potential of invasive plant expansion. Terrestrial represents terrestrial ecoregions; Freshwater represents freshwater ecoregions; Codes used in this figure are defined as follows: For terrestrial ecoregions: BF: Boreal Forests/Taiga; DXS: Deserts and Xeric Shrublands; FGS: Flooded Grasslands and Savannas; IW: Inland Water; MG: Mangroves; MFWS: Mediterranean Forests, Woodlands and Scrub; MGS: Montane Grasslands and Shrublands; RI: Rock and Ice; TBMF: Temperate Broadleaf and Mixed Forests; TCF: Temperate Conifer Forests; TGSS: Temperate Grasslands, Savannas and Shrublands; TSCF: Tropical and Subtropical Coniferous Forests; TSDBF: Tropical and Subtropical Dry Broadleaf Forests; TSGSS: Tropical and Subtropical Grasslands, Savannas and Shrublands; TSMBF: Tropical and Subtropical Moist Broadleaf Forests; TD: Tundra. For freshwater ecoregions: LL: Large Lakes; LRD: Large River Deltas; MF: Montane Freshwaters; OI: Oceanic Islands; PF: Polar Freshwaters; TCR: Temperate Coastal Rivers; TFRW: Temperate Floodplain Rivers and Wetlands; TUR: Temperate Upland Rivers; TSCR: Tropical and Subtropical Coastal Rivers; TSFRWC: Tropical and Subtropical Floodplain Rivers and Wetland Complexes; TSUR: Tropical and Subtropical Upland Rivers; XFEB: Xeric Freshwaters and Endorheic (closed) Basins.

10.7717/peerj.6479/supp-4 Supplemental Information 4 Fig. S2. Map showing the potential for invasive plant expansion in RCPs 4.5 and 8.5. The colors coupled with the numbers in this figure represent the level of IPS expansion potential across different ecoregions. Blue means there is a very high chance of expansion and tan-yellow means a low chance of expansion.

10.7717/peerj.6479/supp-5 Supplemental Information 5 Invasive and native regions of 387 invasive plant species.

10.7717/peerj.6479/supp-6 Supplemental Information 6 Occurrence records of invasive plant species.

## Additional Information and Declarations

### Data Availability

The following information was supplied regarding data availability: The raw data are available in the Supplemental Files. The raw data shows the occurrence records, especially geographic coordinates, from the Global Biodiversity Information Facility (GBIF; www.gbif.org).
