# Supplementary material for: Potential invasive plant expansion in global ecoregions under climate change
Source: PeerJ. 2019 Mar 5;7:e6479. doi: 10.7717/peerj.6479 (PMC6407507; doi:10.7717/peerj.6479)
Supplement: Supplemental Information 1 [file peerj-07-6479-s001.docx]

**Table S1. IPS, AUC values, omission rates and the results of Maxent jackknife test.**

| Name | Type | Record | AUC | Bio1 | Bio2 | Bio4 | Bio5 | Bio6 | Bio12 | Bio13 | Bio14 | Bio15 | Omission rates |
| --- | --- | --- | --- | --- | --- | --- | --- | --- | --- | --- | --- | --- | --- |
| *Abelmoschus moschatus* | Herb | 487 | 0.934 | 1.4 | 6.8 | 15.7 | 1.5 | 16.5 | 11.8 | 44.1 | 1 | 1.2 | 0.010±0.008 |
| *Abrus precatorius* | Shrub | 1085 | 0.927 | 12.7 | 1.6 | 44.8 | 3.4 | 12.3 | 4.6 | 17.4 | 0.4 | 3 | 0.031±0.032 |
| *Acacia farnesiana* | Tree | 1840 | 0.874 | 18.1 | 2.6 | 25.5 | 0.7 | 32.8 | 10.1 | 0.6 | 4 | 5.6 | 0.033±0.075 |
| *Acacia longifolia* | Tree | 854 | 0.955 | 36.1 | 2.8 | 40.7 | 3.5 | 10.9 | 2.6 | 1.8 | 0.5 | 1.1 | 0.031±0.035 |
| *Acacia mangium* | Tree | 204 | 0.954 | 5.2 | 2.5 | 37.4 | 1.9 | 17 | 12.1 | 14.3 | 6.5 | 3.3 | 0.011±0.015 |
| *Acacia mearnsii* | Tree | 603 | 0.964 | 34.3 | 1.3 | 30.3 | 12.1 | 11.1 | 4.1 | 2 | 4.1 | 0.7 | 0.009±0.009 |
| *Acacia melanoxylon* | Tree | 1215 | 0.941 | 25.4 | 0.7 | 37.3 | 10.1 | 10.3 | 1.4 | 0.7 | 13.2 | 0.7 | 0.016±0.018 |
| *Acacia nilotica* | Tree | 665 | 0.93 | 21.7 | 0.3 | 43.3 | 2 | 0.2 | 20.4 | 8.7 | 1.4 | 1.9 | 0.019±0.043 |
| *Acacia retinodes* | Tree | 287 | 0.974 | 37.4 | 1.2 | 27.4 | 0.9 | 25.2 | 3.2 | 2.8 | 0.8 | 1.2 | 0.028±0.031 |
| *Acacia saligna* | Tree | 812 | 0.958 | 35 | 3.9 | 25.3 | 0.1 | 28.2 | 2.3 | 3.7 | 1.4 | 0 | 0.023±0.030 |
| *Acanthophora spicifera* | Alga | 148 | 0.972 | 2.7 | 18.9 | 27.4 | 1 | 40.3 | 5.5 | 2.8 | 0.8 | 0.5 | 0.009±0.011 |
| *Acanthus mollis* | Herb | 340 | 0.975 | 34.1 | 7.3 | 15.4 | 0.4 | 28.1 | 7 | 7.3 | 0.3 | 0.1 | 0.020±0.042 |
| *Acer ginnala* | Tree | 116 | 0.965 | 27.1 | 0.5 | 12.2 | 4 | 16.9 | 1.9 | 0.9 | 36 | 0.5 | 0.027±0.059 |
| *Acer platanoides* | Tree | 6086 | 0.816 | 15.1 | 0.1 | 3.4 | 7.5 | 17.7 | 0.7 | 0.3 | 34.9 | 20.2 | 0.024±0.053 |
| *Acroptilon repens* | Herb | 268 | 0.942 | 38.6 | 14.6 | 30 | 3.8 | 0.7 | 5.3 | 0 | 4.5 | 2.4 | 0.007±0.012 |
| *Adenanthera pavonina* | Tree | 162 | 0.947 | 3.5 | 10.5 | 9.8 | 2.2 | 30.2 | 33.8 | 7.1 | 0.9 | 2 | 0.015±0.014 |
| *Aegilops triuncialis* | Herb | 1412 | 0.931 | 34.1 | 2.8 | 14.5 | 8.5 | 28.1 | 2.9 | 4.2 | 4.5 | 0.4 | 0.006±0.009 |
| *Agapanthus praecox* | Herb | 166 | 0.985 | 16 | 0.2 | 29.1 | 22.1 | 18.1 | 2.1 | 0.9 | 10.7 | 0.7 | 0.013±0.020 |
| *Agave americana* | Herb | 469 | 0.945 | 36.2 | 2.3 | 22.5 | 0.4 | 26 | 4.5 | 5.4 | 0.3 | 2.4 | 0.008±0.008 |
| *Ageratina adenophora* | Shrub | 400 | 0.966 | 37.9 | 0.4 | 36.1 | 7.9 | 8.5 | 1.4 | 4.6 | 2.5 | 0.7 | 0.015±0.007 |
| *Ageratina riparia* | Herb | 193 | 0.981 | 27 | 0.2 | 25.3 | 13.6 | 3.2 | 4.5 | 1.9 | 22.6 | 1.7 | 0.007±0.016 |
| *Ageratum conyzoides* | Herb | 1255 | 0.893 | 10 | 2.5 | 49.4 | 5.4 | 0.8 | 14.2 | 16.3 | 0.6 | 0.6 | 0.030±0.036 |
| *Agrostis capillaris* | Herb | 12510 | 0.725 | 13.4 | 0.2 | 13.3 | 15.3 | 9.7 | 3.2 | 0.1 | 37.4 | 7.5 | 0.023±0.025 |
| *Agrostis gigantea* | Herb | 6806 | 0.791 | 44.9 | 0.4 | 14.8 | 2.7 | 10.4 | 0.1 | 0.6 | 25.5 | 0.7 | 0.015±0.032 |
| *Ailanthus altissima* | Tree | 1478 | 0.917 | 50.3 | 0.2 | 9.3 | 1.6 | 26.7 | 5.6 | 1.5 | 2.6 | 2.3 | 0.020±0.046 |
| *Akebia quinata* | Vine | 153 | 0.98 | 32.4 | 0 | 7.2 | 4.4 | 1.4 | 12.9 | 0.8 | 37.6 | 3.3 | 0.035±0.077 |
| *Albizia julibrissin* | Tree | 273 | 0.959 | 47 | 0.4 | 9.4 | 0.1 | 13.7 | 21.2 | 0.6 | 5.9 | 1.7 | 0.005±0.008 |
| *Albizia lebbeck* | Tree | 403 | 0.931 | 9.2 | 2.4 | 23.2 | 5.2 | 35.5 | 0.3 | 15.3 | 5 | 3.9 | 0.009±0.007 |
| *Aleurites moluccana* | Tree | 115 | 0.959 | 9.9 | 12.1 | 39.1 | 11.7 | 3.4 | 10.8 | 7.3 | 4.5 | 1.2 | 0.031±0.011 |
| *Alliaria petiolata* | Herb | 6134 | 0.814 | 25.1 | 1.2 | 3.8 | 0.1 | 24 | 6.5 | 0.5 | 17.7 | 21.2 | 0.019±0.009 |
| *Alnus glutinosa* | Tree | 8816 | 0.772 | 23.8 | 5.5 | 11.2 | 0.7 | 12.4 | 5 | 0.2 | 26.9 | 14.2 | 0.021±0.027 |
| *Alternanthera philoxeroides* | Aquatic Plant | 232 | 0.959 | 34.2 | 0.3 | 15.7 | 1.3 | 5.1 | 0.1 | 2.6 | 40.3 | 0.4 | 0.008±0.017 |
| *Alternanthera sessilis* | Herb | 632 | 0.894 | 19.3 | 4.2 | 2.9 | 1.6 | 36.5 | 3.8 | 30.9 | 0.5 | 0.4 | 0.010±0.012 |
| *Ambrosia artemisiifolia* | Herb | 2615 | 0.869 | 48.1 | 0.1 | 3.4 | 1.1 | 0.2 | 4.5 | 5.3 | 35.5 | 1.9 | 0.010±0.023 |
| *Ammophila arenaria* | Herb | 1774 | 0.923 | 28.8 | 19.3 | 22.9 | 7.2 | 16.7 | 0.2 | 1.1 | 2.6 | 1.1 | 0.012±0.015 |
| *Ampelopsis brevipedunculata* | Vine | 202 | 0.974 | 13.6 | 2.7 | 20.2 | 10.4 | 0.8 | 43.6 | 0.7 | 6.8 | 1.2 | 0.018±0.031 |
| *Andropogon gayanus* | Herb | 541 | 0.942 | 16 | 0.6 | 63 | 0.5 | 7.5 | 3.4 | 5.8 | 0.7 | 2.5 | 0.009±0.010 |
| *Andropogon virginicus* | Herb | 611 | 0.951 | 31.6 | 0.8 | 5.7 | 9.2 | 2.9 | 8.5 | 16.2 | 20.4 | 4.6 | 0.009±0.011 |
| *Angiopteris evecta* | Fern | 100 | 0.949 | 12.1 | 2.8 | 21.3 | 6.1 | 4.1 | 11.6 | 31.4 | 10 | 0.7 | 0.024±0.019 |
| *Annona glabra* | Tree | 288 | 0.958 | 2.2 | 4.7 | 16.9 | 5.6 | 42.4 | 12.2 | 12.3 | 2.8 | 0.8 | 0.022±0.027 |
| *Annona squamosa* | Tree | 184 | 0.939 | 0.9 | 1.4 | 25.5 | 2.1 | 36.6 | 13.9 | 13.1 | 3.5 | 3 | 0.009±0.021 |
| *Anredera cordifolia* | Vine | 384 | 0.957 | 38.1 | 0.6 | 35.5 | 7.7 | 4 | 2.4 | 5 | 5.9 | 0.8 | 0.019±0.032 |
| *Antigonon leptopus* | Vine | 410 | 0.923 | 11.9 | 2 | 37.4 | 2.2 | 29.8 | 4.8 | 4.1 | 2.8 | 5 | 0.025±0.018 |
| *Archontophoenix cunninghamiana* | Tree | 177 | 0.986 | 11.7 | 0.2 | 38.9 | 11.6 | 9.3 | 0.4 | 2.3 | 25.1 | 0.5 | 0.020±0.045 |
| *Ardisia crenata* | Shrub | 236 | 0.972 | 12.6 | 5.3 | 16.3 | 1.1 | 0.1 | 35.7 | 19.7 | 8.1 | 1.2 | 0.013±0.030 |
| *Arundo donax* | Herb | 1292 | 0.897 | 44.5 | 3.2 | 20.5 | 0.7 | 20.5 | 8.4 | 0.4 | 0.2 | 1.8 | 0.019±0.042 |
| *Asparagus densiflorus* | Herb | 126 | 0.95 | 11.7 | 0.3 | 35.6 | 20.4 | 9 | 6.2 | 10 | 2.1 | 4.6 | 0.024±0.018 |
| *Asparagus officinalis* | Herb | 3737 | 0.858 | 39.3 | 0.1 | 3.2 | 1.9 | 24.1 | 3.2 | 3.1 | 4 | 21.2 | 0.032±0.034 |
| *Asystasia gangetica* | Herb | 534 | 0.928 | 6.7 | 5.7 | 53.9 | 3.8 | 18 | 4.2 | 4.8 | 2.7 | 0.3 | 0.020±0.020 |
| *Austroeupatorium inulifolium* | Herb | 119 | 0.979 | 13.4 | 2.8 | 56.6 | 3.7 | 0 | 0.2 | 8.3 | 14.2 | 0.8 | 0.004±0.006 |
| *Azolla pinnata* | Aquatic Plant | 384 | 0.946 | 17.6 | 2 | 46.3 | 5.7 | 9.4 | 8.3 | 0.4 | 4.7 | 5.5 | 0.020±0.022 |
| *Bacopa monnieri* | Herb | 601 | 0.911 | 2.6 | 1.3 | 23.8 | 7.4 | 48.7 | 0.6 | 5.8 | 7.8 | 2.1 | 0.004±0.009 |
| *Bambusa vulgaris* | Herb | 154 | 0.915 | 13.6 | 3.1 | 47.6 | 1.5 | 7.3 | 12.4 | 7 | 6.7 | 0.8 | 0.003±0.006 |
| *Begonia cucullata* | Herb | 137 | 0.943 | 28.3 | 2.2 | 23.6 | 0.2 | 5.8 | 29.5 | 1.1 | 9.1 | 0.4 | 0.032±0.072 |
| *Bellis perennis* | Herb | 7734 | 0.788 | 26.1 | 4.3 | 21.6 | 0.8 | 15 | 5.3 | 0.5 | 9.7 | 16.8 | 0.018±0.020 |
| *Berberis darwinii* | Tree | 317 | 0.982 | 26.5 | 0.1 | 24.5 | 14.9 | 14 | 1.4 | 0 | 18.2 | 0.4 | 0.004±0.006 |
| *Berberis thunbergii* | Shrub | 898 | 0.948 | 32.4 | 3.1 | 5.9 | 0.9 | 14.6 | 2.2 | 0.1 | 39.3 | 1.5 | 0.038±0.032 |
| *Bidens pilosa* | Herb | 2380 | 0.853 | 17.3 | 0.1 | 25.2 | 5.6 | 33.1 | 14.5 | 0.5 | 2.8 | 0.8 | 0.006±0.009 |
| *Bothriochloa pertusa* | Herb | 333 | 0.932 | 19.2 | 1.5 | 33.6 | 0.8 | 11 | 10.2 | 14.5 | 7.3 | 1.9 | 0.009±0.009 |
| *Brassica elongata* | Herb | 211 | 0.965 | 25.6 | 5.6 | 22.1 | 3.2 | 14.5 | 3.1 | 6 | 4.6 | 15.3 | 0.006±0.008 |
| *Brassica tournefortii* | Herb | 1726 | 0.914 | 13.7 | 0.1 | 4.7 | 0.2 | 59.2 | 5.4 | 14 | 0.6 | 1.9 | 0.022±0.048 |
| *Bromus inermis* | Herb | 4373 | 0.831 | 47.1 | 0.7 | 13.7 | 2 | 14.2 | 1.8 | 1.5 | 12.7 | 6.4 | 0.026±0.027 |
| *Bromus rubens* | Herb | 2721 | 0.889 | 38.5 | 0.1 | 9.4 | 1.6 | 38.5 | 8 | 2.2 | 0.8 | 1 | 0.042±0.047 |
| *Bromus tectorum* | Herb | 4780 | 0.826 | 51.9 | 0.1 | 27.4 | 1.8 | 17 | 0.6 | 0.5 | 0.4 | 0.2 | 0.020±0.026 |
| *Buddleja davidii* | Shrub | 2454 | 0.901 | 22.3 | 0.3 | 1.3 | 10 | 19.7 | 0.7 | 0.2 | 38.6 | 6.8 | 0.012±0.014 |
| *Buddleja madagascariensis* | Vine | 113 | 0.981 | 10.5 | 0.7 | 33.1 | 19.3 | 18.4 | 8.7 | 0.7 | 8 | 0.7 | 0.008±0.017 |
| *Butomus umbellatus* | Aquatic Plant | 3222 | 0.881 | 7.1 | 12.3 | 6.6 | 15.6 | 7.1 | 0.5 | 2 | 17.2 | 31.5 | 0.014±0.012 |
| *Cabomba caroliniana* | Aquatic Plant | 219 | 0.962 | 20.6 | 3.3 | 16.6 | 2.2 | 0 | 13.8 | 2.3 | 39.4 | 1.8 | 0.005±0.007 |
| *Caesalpinia decapetala* | Tree | 256 | 0.961 | 33.8 | 0.3 | 2.5 | 15.1 | 4 | 16.4 | 18 | 5.1 | 4.7 | 0.020±0.045 |
| *Calluna vulgaris* | Herb | 12650 | 0.724 | 21.2 | 7.8 | 18.8 | 0.7 | 3.7 | 5.4 | 0.3 | 30 | 12.1 | 0.008±0.012 |
| *Camelina sativa* | Herb | 1553 | 0.923 | 23.6 | 0.2 | 7.7 | 11 | 10 | 2.4 | 2.2 | 18.6 | 24.3 | 0.022±0.021 |
| *Campuloclinium macrocephalum* | Herb | 152 | 0.978 | 14.4 | 2.9 | 38.9 | 2.3 | 3.7 | 10.4 | 16.5 | 8.4 | 2.6 | 0.022±0.022 |
| *Canna indica* | Herb | 679 | 0.908 | 19.1 | 0.4 | 37.7 | 2.2 | 2 | 25.5 | 0.3 | 12.6 | 0.2 | 0.032±0.047 |
| *Cardamine flexuosa* | Herb | 5508 | 0.824 | 10.5 | 4.6 | 15.3 | 11.5 | 14.7 | 0.9 | 0 | 42.4 | 0 | 0.007±0.009 |
| *Cardiospermum grandiflorum* | Vine | 432 | 0.936 | 11.2 | 0.2 | 42.3 | 2.1 | 15.5 | 9.7 | 3.5 | 8.5 | 6.9 | 0.009±0.009 |
| *Carduus nutans* | Herb | 3902 | 0.861 | 32.6 | 0.3 | 5.5 | 1.3 | 20.2 | 2.6 | 2.4 | 15.4 | 19.7 | 0.020±0.017 |
| *Carpobrotus edulis* | Succulent | 458 | 0.969 | 37.4 | 6.1 | 27.9 | 0.5 | 23.4 | 0.9 | 3.1 | 0.1 | 0.6 | 0.009±0.015 |
| *Castilla elastica* | Tree | 196 | 0.962 | 3.1 | 1.9 | 30.3 | 0.3 | 23.2 | 30 | 2.6 | 4.1 | 4.4 | 0.018±0.023 |
| *Casuarina equisetifolia* | Tree | 560 | 0.936 | 6.7 | 22.7 | 32.3 | 4.1 | 25.7 | 5.7 | 1.5 | 0.2 | 1 | 0.062±0.138 |
| *Caulerpa taxifolia* | Alga | 120 | 0.975 | 3.2 | 24 | 42.3 | 5.8 | 14.9 | 2.5 | 3.6 | 0.7 | 3.1 | 0.031±0.035 |
| *Cecropia peltata* | Tree | 368 | 0.949 | 0.5 | 4.3 | 50.8 | 0.3 | 12.4 | 21.9 | 1.4 | 3.3 | 5.1 | 0.011±0.017 |
| *Cecropia schreberiana* | Tree | 382 | 0.948 | 2 | 4.6 | 53.3 | 0.4 | 9.6 | 18.4 | 1.3 | 4.9 | 5.4 | 0.026±0.058 |
| *Cedrela odorata* | Tree | 520 | 0.935 | 3.5 | 2.3 | 61.5 | 1.7 | 6.4 | 11.1 | 3.5 | 9.5 | 0.4 | 0.013±0.029 |
| *Celastrus orbiculatus* | Vine | 329 | 0.974 | 30.2 | 0.2 | 11.4 | 2.8 | 1.2 | 18.3 | 0 | 35.5 | 0.3 | 0.014±0.011 |
| *Cenchrus ciliaris* | Herb | 1881 | 0.877 | 35 | 0 | 13.9 | 1.3 | 19.6 | 25.6 | 0.4 | 2.7 | 1.6 | 0.016±0.037 |
| *Cenchrus echinatus* | Herb | 1182 | 0.875 | 53.6 | 1.2 | 22.7 | 2.8 | 1.9 | 8.7 | 0.7 | 6.9 | 1.5 | 0.017±0.022 |
| *Cenchrus polystachios* | Herb | 105 | 0.936 | 3.7 | 2.1 | 44.9 | 0.4 | 23.7 | 1.1 | 15.9 | 0.9 | 7.2 | 0.016±0.023 |
| *Centaurea diffusa* | Herb | 308 | 0.964 | 36.6 | 0.5 | 29.6 | 8.7 | 19.5 | 0.1 | 2 | 1.6 | 1.4 | 0.021±0.025 |
| *Centaurea melitensis* | Herb | 2616 | 0.883 | 40.7 | 0.4 | 26.1 | 0.1 | 21.4 | 8 | 1.2 | 0.2 | 1.8 | 0.019±0.019 |
| *Centaurea solstitialis* | Herb | 1450 | 0.922 | 41.8 | 0.1 | 26.4 | 1.3 | 20.7 | 5.1 | 0.8 | 1.5 | 2.2 | 0.008±0.010 |
| *Cerastium fontanum* | Herb | 11820 | 0.729 | 32.1 | 0.5 | 18.2 | 0.3 | 8.7 | 5.2 | 0.1 | 34.2 | 0.7 | 0.013±0.029 |
| *Ceratophyllum demersum* | Aquatic Plant | 2077 | 0.843 | 48.5 | 3.4 | 8.5 | 7.5 | 0.4 | 26.9 | 0.1 | 0.1 | 4.6 | 0.014±0.031 |
| *Cestrum nocturnum* | Shrub | 440 | 0.945 | 14.6 | 0.3 | 45.1 | 3.6 | 4.1 | 17.1 | 1.8 | 10.7 | 2.7 | 0.015±0.033 |
| *Cestrum parqui* | Shrub | 401 | 0.96 | 21.9 | 1.8 | 47.4 | 2.2 | 7.4 | 5.7 | 8.5 | 4.5 | 0.7 | 0.009±0.012 |
| *Chromolaena odorata* | Herb | 678 | 0.914 | 0.4 | 2.5 | 46.2 | 4.2 | 12.3 | 13.9 | 15.6 | 4.8 | 0.1 | 0.030±0.013 |
| *Chrysanthemoides monilifera* | Herb | 345 | 0.977 | 33.2 | 2.7 | 36.2 | 0.1 | 16.4 | 2.4 | 2.1 | 6.4 | 0.5 | 0.016±0.025 |
| *Chrysobalanus icaco* | Tree | 510 | 0.954 | 5.2 | 2.1 | 32.4 | 0.4 | 38.2 | 18.7 | 0.8 | 1.3 | 0.8 | 0.029±0.039 |
| *Cinchona pubescens* | Tree | 226 | 0.976 | 6 | 0.9 | 59.7 | 16.6 | 5.5 | 4.3 | 0.3 | 6.2 | 0.4 | 0.008±0.015 |
| *Cinnamomum camphora* | Tree | 421 | 0.962 | 29.9 | 1.3 | 13 | 10 | 7.5 | 12.4 | 3.5 | 19.8 | 2.6 | 0.032±0.037 |
| *Cirsium arvense* | Herb | 10130 | 0.749 | 37.5 | 0.1 | 14.5 | 0.7 | 11.6 | 0.8 | 0.4 | 5.9 | 28.5 | 0.038±0.086 |
| *Cirsium vulgare* | Herb | 11154 | 0.735 | 31.8 | 0.1 | 8.2 | 0.5 | 35.3 | 6.5 | 0.1 | 10.5 | 6.9 | 0.016±0.015 |
| *Clematis terniflora* | Vine | 366 | 0.969 | 23 | 1.7 | 13 | 0.2 | 10.4 | 22.5 | 0.9 | 28 | 0.4 | 0.006±0.014 |
| *Clematis vitalba* | Vine | 4557 | 0.849 | 24.4 | 6.1 | 25.1 | 1.7 | 15.5 | 0.7 | 1.5 | 1.8 | 23.1 | 0.004±0.005 |
| *Clidemia hirta* | Shrub | 618 | 0.944 | 1.4 | 0.6 | 56.3 | 0 | 6.3 | 18.3 | 0.6 | 15.9 | 0.7 | 0.017±0.022 |
| *Coccinia grandis* | Vine | 154 | 0.93 | 3.8 | 5.5 | 53.9 | 1.2 | 27.3 | 3.6 | 1.5 | 2.9 | 0.3 | 0.010±0.016 |
| *Colubrina asiatica* | Shrub | 271 | 0.969 | 0.8 | 21.3 | 22.4 | 0.4 | 28 | 20.1 | 3 | 0.4 | 3.6 | 0.016±0.009 |
| *Commelina benghalensis* | Herb | 579 | 0.905 | 29.7 | 0.6 | 21.3 | 8.5 | 19.8 | 14.1 | 3.2 | 1.3 | 1.4 | 0.013±0.009 |
| *Coronilla varia* | Herb | 359 | 0.952 | 38.1 | 2.8 | 15.1 | 4 | 15.9 | 6.8 | 3.3 | 12.8 | 1.1 | 0.025±0.035 |
| *Cortaderia jubata* | Herb | 149 | 0.979 | 34.6 | 2.1 | 39.6 | 12.8 | 5.1 | 0.1 | 3.7 | 0.6 | 1.5 | 0.011±0.024 |
| *Cortaderia selloana* | Herb | 681 | 0.952 | 37.1 | 1.5 | 28.1 | 5.1 | 23.4 | 0 | 4.3 | 0.1 | 0.4 | 0.008±0.014 |
| *Cotinus coggygria* | Tree | 228 | 0.964 | 18.9 | 5.8 | 23.3 | 2.3 | 26.2 | 22.6 | 0.1 | 0.2 | 0.7 | 0.027±0.061 |
| *Crassula helmsii* | Aquatic Plant | 931 | 0.956 | 24.5 | 0.4 | 23.7 | 0.8 | 17.6 | 0.3 | 2.4 | 17.5 | 12.9 | 0.010±0.022 |
| *Cryptostegia grandiflora* | Vine | 315 | 0.958 | 25.5 | 0.5 | 38.5 | 3.9 | 1.2 | 9.7 | 6.9 | 10 | 3.9 | 0.011±0.024 |
| *Cryptostegia madagascariensis* | Vine | 155 | 0.961 | 5.9 | 0.6 | 24.8 | 7.3 | 30.6 | 1.9 | 17.8 | 2.8 | 8.3 | 0.031±0.068 |
| *Cupaniopsis anacardioides* | Tree | 550 | 0.967 | 7.6 | 6 | 51.6 | 5.3 | 9.3 | 13.5 | 1.9 | 3.1 | 1.7 | 0.015±0.011 |
| *Cyathea cooperi* | Fern | 255 | 0.975 | 14.5 | 0.5 | 38.5 | 8.5 | 8 | 13.2 | 0.2 | 15.4 | 1.2 | 0.029±0.066 |
| *Cynara cardunculus* | Herb | 464 | 0.966 | 39.7 | 2.9 | 29.8 | 0.3 | 15.7 | 4.2 | 6.6 | 0.4 | 0.3 | 0.008±0.019 |
| *Cynodon dactylon* | Herb | 5921 | 0.774 | 25.3 | 0.1 | 14.9 | 0.8 | 50.6 | 6.3 | 0.2 | 1.6 | 0.2 | 0.018±0.013 |
| *Cynoglossum officinale* | Herb | 2842 | 0.888 | 32.6 | 0.2 | 15.3 | 0.9 | 10.7 | 3.3 | 1.8 | 10.3 | 24.8 | 0.019±0.041 |
| *Cyperus rotundus* | Herb | 2336 | 0.834 | 37.7 | 1 | 11.2 | 0.3 | 31.7 | 13.4 | 1 | 2 | 1.7 | 0.010±0.015 |
| *Cytisus scoparius* | Shrub | 6561 | 0.808 | 31.5 | 0.9 | 20.1 | 0.5 | 22.2 | 4.7 | 0.1 | 16.5 | 3.6 | 0.004±0.006 |
| *Cytisus striatus* | Shrub | 339 | 0.978 | 27.3 | 8.4 | 30.4 | 0.6 | 23.2 | 4.9 | 2.8 | 2 | 0.3 | 0.058±0.130 |
| *Delairea odorata* | Vine | 346 | 0.975 | 38.8 | 0.4 | 30.6 | 7.7 | 17.5 | 2.5 | 1.3 | 0.3 | 1 | 0.050±0.112 |
| *Dichrostachys cinerea* | Tree | 691 | 0.93 | 15.9 | 0.2 | 58.5 | 1.3 | 0.5 | 14.5 | 1.8 | 2.6 | 4.7 | 0.010±0.022 |
| *Dioscorea bulbifera* | Herb | 682 | 0.93 | 10.5 | 1.6 | 9.3 | 2.7 | 7.3 | 6.4 | 60 | 0 | 2.1 | 0.011±0.009 |
| *Dipogon lignosus* | Vine | 272 | 0.975 | 31 | 0.1 | 35.5 | 3 | 21.6 | 1.5 | 0 | 6.6 | 0.6 | 0.013±0.029 |
| *Duchesnea indica* | Herb | 786 | 0.942 | 34.2 | 1.1 | 1.4 | 4.3 | 26.4 | 16.8 | 0.3 | 15.1 | 0.3 | 0.017±0.021 |
| *Egeria densa* | Aquatic Plant | 375 | 0.935 | 27.8 | 0.2 | 6.2 | 3.4 | 29.6 | 11.5 | 1.2 | 19.2 | 0.9 | 0.011±0.014 |
| *Eichhornia crassipes* | Aquatic Plant | 809 | 0.885 | 24.7 | 1.1 | 7.5 | 3 | 27.4 | 25.3 | 3.1 | 7.2 | 0.8 | 0.025±0.030 |
| *Elaeagnus angustifolia* | Tree | 669 | 0.921 | 47.8 | 0.3 | 31.3 | 1 | 14 | 1.9 | 0.4 | 1.6 | 1.8 | 0.003±0.006 |
| *Elaeagnus pungens* | Shrub | 101 | 0.979 | 29.5 | 0.3 | 14.4 | 0.2 | 8.9 | 14.9 | 2.2 | 27.8 | 1.7 | 0.009±0.021 |
| *Elaeagnus umbellata* | Tree | 409 | 0.962 | 42.7 | 0.6 | 3.8 | 1.8 | 7.4 | 32.2 | 0.2 | 10.4 | 0.8 | 0.017±0.010 |
| *Elaeis guineensis* | Palm | 139 | 0.964 | 2.9 | 0.5 | 15.6 | 1.2 | 52.4 | 22.5 | 0.9 | 0.2 | 3.9 | 0.007±0.011 |
| *Elephantopus mollis* | Herb | 789 | 0.924 | 8 | 0.6 | 42.6 | 1 | 1.3 | 19.5 | 24.5 | 2 | 0.6 | 0.010±0.011 |
| *Elodea canadensis* | Aquatic Plant | 1250 | 0.917 | 49.3 | 0.2 | 10.1 | 5.1 | 0 | 6.1 | 0.2 | 19.4 | 9.7 | 0.015±0.017 |
| *Epipremnum pinnatum* | Vine | 183 | 0.954 | 1.2 | 12.9 | 5.1 | 16.1 | 4.1 | 17.1 | 33.7 | 7.1 | 2.8 | 0.022±0.013 |
| *Erigeron karvinskianus* | Herb | 1035 | 0.93 | 24.7 | 0.2 | 13.1 | 18.3 | 29.7 | 12.4 | 0 | 1.4 | 0.1 | 0.040±0.048 |
| *Eriobotrya japonica* | Tree | 305 | 0.944 | 36.9 | 3.8 | 0.7 | 12.1 | 28.3 | 12.1 | 1.9 | 2.1 | 2.2 | 0.018±0.019 |
| *Erodium cicutarium* | Herb | 9467 | 0.751 | 37.1 | 0.2 | 22.3 | 1.2 | 34.1 | 1.3 | 2.2 | 0.2 | 1.3 | 0.010±0.016 |
| *Eugenia uniflora* | Tree | 273 | 0.941 | 21.8 | 0.3 | 19 | 5.9 | 1.4 | 23.3 | 14.1 | 14 | 0.2 | 0.022±0.007 |
| *Euonymus fortunei* | Vine | 185 | 0.971 | 28.4 | 0.3 | 5.7 | 0.3 | 23.3 | 10.8 | 1.9 | 28.3 | 1 | 0.009±0.009 |
| *Eupatorium cannabinum* | Herb | 5649 | 0.826 | 19.6 | 3.6 | 19.8 | 0.8 | 10.2 | 0.3 | 0.6 | 18.3 | 26.8 | 0.013±0.029 |
| *Euphorbia esula* | Herb | 2700 | 0.889 | 33.9 | 0.1 | 11.8 | 0.7 | 7.7 | 3.8 | 2.4 | 11.7 | 27.9 | 0.028±0.023 |
| *Ficus rubiginosa* | Tree | 673 | 0.962 | 8 | 0.8 | 53 | 4.4 | 5.7 | 10.4 | 3.3 | 13.4 | 0.8 | 0.016±0.020 |
| *Flacourtia indica* | Tree | 264 | 0.932 | 18.2 | 0.7 | 19 | 7.3 | 14 | 6.2 | 25.7 | 1.3 | 7.6 | 0.011±0.010 |
| *Flemingia strobilifera* | Shrub | 121 | 0.959 | 6.4 | 13.8 | 10.8 | 0.9 | 0 | 20.7 | 42.5 | 4.2 | 0.7 | 0.023±0.013 |
| *Fragaria vesca* | Herb | 10477 | 0.746 | 20.8 | 0 | 11.1 | 14.7 | 16.4 | 6.1 | 0.1 | 30.4 | 0.3 | 0.019±0.042 |
| *Frangula alnus* | Shrub | 5668 | 0.823 | 16.7 | 2.8 | 9.1 | 8.4 | 13.5 | 0.9 | 0.2 | 35 | 13.4 | 0.015±0.023 |
| *Fuchsia boliviana* | Tree | 104 | 0.982 | 23.9 | 0.2 | 47.2 | 6.8 | 4.5 | 0.6 | 8.9 | 4.1 | 3.7 | 0.012±0.026 |
| *Fuchsia magellanica* | Vine | 866 | 0.96 | 11.9 | 6.1 | 23.3 | 22.2 | 12.3 | 0.9 | 0 | 23.1 | 0.2 | 0.022±0.026 |
| *Genista monspessulana* | Shrub | 652 | 0.958 | 45.4 | 0.2 | 30 | 0.2 | 15.7 | 5.4 | 0.7 | 0.7 | 1.7 | 0.006±0.009 |
| *Glyceria maxima* | Aquatic Plant | 4595 | 0.848 | 10.2 | 0.4 | 3.9 | 20 | 10.1 | 0.2 | 0.1 | 33.1 | 22 | 0.021±0.025 |
| *Grevillea robusta* | Tree | 302 | 0.956 | 30.4 | 0.6 | 17.1 | 9.2 | 22.6 | 3.2 | 1 | 13.1 | 2.9 | 0.038±0.086 |
| *Gunnera tinctoria* | Herb | 259 | 0.986 | 12.3 | 7.6 | 15.8 | 29.9 | 13.6 | 0.9 | 0 | 19.8 | 0 | 0.035±0.078 |
| *Haematoxylum campechianum* | Tree | 224 | 0.979 | 1.8 | 0.3 | 30.8 | 1.4 | 43.7 | 0.6 | 6 | 15.1 | 0.3 | 0.042±0.094 |
| *Hedera helix* | Vine | 7543 | 0.792 | 28.7 | 5.2 | 10.2 | 0.4 | 32.8 | 3.5 | 0.6 | 5.6 | 13.1 | 0.063±0.141 |
| *Hedychium coronarium* | Herb | 312 | 0.947 | 11.1 | 0.9 | 20 | 4.5 | 4 | 49.7 | 3.9 | 5.8 | 0.2 | 0.031±0.038 |
| *Hedychium flavescens* | Herb | 121 | 0.972 | 9.8 | 0.7 | 35 | 23.6 | 7.9 | 4.9 | 1.3 | 16.1 | 0.7 | 0.016±0.012 |
| *Hedychium gardnerianum* | Herb | 105 | 0.978 | 10.4 | 1.1 | 30.7 | 18.8 | 11 | 11.4 | 0 | 16.3 | 0.3 | 0.008±0.017 |
| *Heliotropium curassavicum* | Herb | 1657 | 0.87 | 33.9 | 2.7 | 15 | 6.2 | 13.8 | 15.7 | 2.8 | 5.3 | 4.6 | 0.030±0.066 |
| *Heracleum mantegazzianum* | Herb | 3299 | 0.88 | 10 | 7.5 | 2.7 | 15.1 | 15 | 0.2 | 0 | 30.8 | 18.6 | 0.033±0.022 |
| *Heteropogon contortus* | Herb | 2620 | 0.857 | 18.6 | 0.6 | 36.2 | 3.4 | 18.3 | 11.5 | 5.4 | 1.3 | 4.6 | 0.015±0.006 |
| *Hieracium aurantiacum* | Herb | 1563 | 0.927 | 24.5 | 0.1 | 6.5 | 6.3 | 12.5 | 0.1 | 0.4 | 49.5 | 0.1 | 0.023±0.052 |
| *Hieracium floribundum* | Herb | 195 | 0.978 | 27.1 | 4.5 | 7.2 | 1.7 | 8.8 | 7.3 | 0.2 | 37.2 | 5.9 | 0.006±0.014 |
| *Hieracium pilosella* | Herb | 5081 | 0.835 | 21.9 | 3.5 | 22.7 | 0.2 | 10.9 | 0.7 | 0.2 | 16.8 | 23.1 | 0.013±0.009 |
| *Holcus lanatus* | Herb | 9493 | 0.76 | 38.3 | 0.7 | 20.8 | 0.2 | 29.5 | 4.4 | 0.4 | 4.9 | 0.9 | 0.014±0.018 |
| *Houttuynia cordata* | Shrub | 209 | 0.959 | 29.3 | 9.2 | 11.7 | 1 | 7.5 | 27.2 | 11.3 | 1 | 1.8 | 0.005±0.007 |
| *Hydrilla verticillata* | Aquatic Plant | 551 | 0.916 | 32.5 | 1.1 | 18.8 | 1.1 | 5.2 | 28.2 | 6 | 5.5 | 1.6 | 0.016±0.021 |
| *Hydrocharis morsus-ranae* | Aquatic Plant | 2774 | 0.895 | 9.6 | 7.4 | 2.1 | 16.7 | 8.4 | 0.8 | 4.4 | 25.5 | 25.1 | 0.003±0.005 |
| *Hypericum perforatum* | Herb | 8984 | 0.765 | 36.8 | 0.2 | 10.4 | 0.2 | 28.6 | 9.2 | 0.6 | 9 | 4.9 | 0.010±0.007 |
| *Hypnea musciformis* | Alga | 103 | 0.963 | 22.2 | 22 | 21.6 | 10.5 | 10.4 | 1.7 | 7.4 | 0.2 | 4.1 | 0.020±0.020 |
| *Hypochaeris radicata* | Herb | 9667 | 0.758 | 35.9 | 0.7 | 23 | 0.1 | 31.3 | 2.7 | 0 | 4.5 | 1.8 | 0.012±0.013 |
| *Impatiens glandulifera* | Herb | 4730 | 0.843 | 4.9 | 5.1 | 5 | 22.3 | 15.4 | 0 | 0.1 | 36.9 | 10.4 | 0.024±0.029 |
| *Impatiens walleriana* | Herb | 266 | 0.947 | 12.8 | 1.5 | 31.5 | 7.2 | 6.7 | 28.6 | 1 | 9.8 | 0.8 | 0.011±0.006 |
| *Imperata cylindrica* | Herb | 2106 | 0.867 | 37.6 | 2.2 | 8.1 | 10 | 24.1 | 15.8 | 0.1 | 0.3 | 1.8 | 0.011±0.009 |
| *Ipomoea aquatica* | Vine | 373 | 0.926 | 29 | 1.8 | 25 | 2.3 | 19.8 | 2.8 | 16.2 | 0.1 | 3 | 0.005±0.007 |
| *Ipomoea cairica* | Vine | 524 | 0.924 | 13.8 | 1 | 34.5 | 7.4 | 30.4 | 2 | 3.6 | 6.9 | 0.3 | 0.007±0.008 |
| *Iris pseudacorus* | Herb | 8026 | 0.783 | 24.3 | 3.8 | 7.2 | 0.6 | 17.8 | 4.3 | 0.5 | 19.6 | 21.9 | 0.032±0.011 |
| *Ischaemum polystachyum* | Herb | 210 | 0.962 | 12.2 | 0.6 | 47.6 | 17.4 | 1.5 | 4.8 | 9.5 | 5.6 | 0.9 | 0.030±0.067 |
| *Jatropha gossypiifolia* | Herb | 406 | 0.925 | 2.4 | 4.3 | 35.3 | 2.2 | 40.7 | 4.1 | 8.7 | 0.4 | 1.8 | 0.055±0.107 |
| *Juncus tenuis* | Herb | 5460 | 0.82 | 42.3 | 0.2 | 2.5 | 1.4 | 0.1 | 11.5 | 0.4 | 36.8 | 4.8 | 0.010±0.013 |
| *Kalanchoe pinnata* | Succulent | 386 | 0.941 | 0.8 | 0.3 | 28.1 | 5.9 | 22.1 | 25.2 | 0.8 | 16.4 | 0.3 | 0.013±0.015 |
| *Lagarosiphon major* | Aquatic Plant | 548 | 0.972 | 17 | 0.4 | 13.8 | 21.7 | 17.3 | 2.2 | 2 | 12.2 | 13.4 | 0.012±0.010 |
| *Landoltia punctata* | Aquatic Plant | 107 | 0.957 | 36.6 | 4.7 | 22.9 | 1.4 | 14.5 | 11.1 | 2.1 | 1.8 | 4.9 | 0.009±0.012 |
| *Lantana camara* | Shrub | 2357 | 0.858 | 13.9 | 0.2 | 26.7 | 4.9 | 33.3 | 10.3 | 2.1 | 7.9 | 0.6 | 0.034±0.032 |
| *Launaea intybacea* | Shrub | 526 | 0.938 | 16.6 | 2.6 | 40.7 | 4.9 | 6.7 | 14.7 | 2.8 | 8.7 | 2.3 | 0.023±0.024 |
| *Lepidium latifolium* | Herb | 1103 | 0.926 | 43.6 | 0.5 | 39.5 | 5.3 | 4 | 2.1 | 2.2 | 1.3 | 1.6 | 0.009±0.009 |
| *Lepidium virginicum* | Herb | 2460 | 0.862 | 22.9 | 0.6 | 7.2 | 5.3 | 39.2 | 8.2 | 1.3 | 14.5 | 0.6 | 0.007±0.016 |
| *Lespedeza cuneata* | Herb | 393 | 0.971 | 21.8 | 0.7 | 22.3 | 0.8 | 2.5 | 37 | 6.7 | 7.5 | 0.7 | 0.039±0.087 |
| *Leucaena leucocephala* | Tree | 950 | 0.896 | 2 | 1.4 | 28.9 | 1.7 | 49.8 | 2.3 | 1.8 | 9.4 | 2.8 | 0.029±0.064 |
| *Ligustrum lucidum* | Tree | 442 | 0.951 | 39.5 | 0.5 | 2.1 | 2.6 | 29.8 | 11.5 | 1.6 | 10.9 | 1.5 | 0.019±0.025 |
| *Ligustrum sinense* | Tree | 486 | 0.962 | 41.7 | 0.3 | 5.1 | 2.9 | 4.2 | 17.5 | 0.3 | 27.6 | 0.4 | 0.029±0.065 |
| *Ligustrum vulgare* | Shrub | 5653 | 0.826 | 21.7 | 2.3 | 8.5 | 0.5 | 25.7 | 1 | 0.4 | 12.5 | 27.5 | 0.020±0.016 |
| *Limnocharis flava* | Aquatic Plant | 191 | 0.935 | 0.7 | 6.4 | 1.9 | 0.3 | 52.7 | 22.4 | 11.1 | 2.7 | 1.9 | 0.007±0.007 |
| *Linaria vulgaris* | Herb | 8370 | 0.774 | 25.9 | 0.1 | 10.3 | 4.9 | 8.6 | 0.1 | 1 | 37.4 | 11.7 | 0.017±0.038 |
| *Litsea glutinosa* | Tree | 497 | 0.966 | 4.5 | 2 | 7 | 2.6 | 7 | 28 | 28.1 | 1 | 19.8 | 0.013±0.027 |
| *Lonicera japonica* | Vine | 1183 | 0.922 | 59.9 | 0.8 | 3.1 | 0.1 | 4.9 | 8.8 | 1.8 | 19 | 1.5 | 0.023±0.020 |
| *Lonicera maackii* | Shrub | 132 | 0.979 | 19.4 | 2.9 | 9.2 | 7.8 | 21.7 | 25.8 | 11 | 1.8 | 0.4 | 0.021±0.019 |
| *Lotus corniculatus* | Herb | 10969 | 0.74 | 34.6 | 1.5 | 15.6 | 0.3 | 23.7 | 4.4 | 0.5 | 13.4 | 5.9 | 0.008±0.012 |
| *Ludwigia peruviana* | Aquatic Plant | 336 | 0.948 | 12 | 2.5 | 41.1 | 2.4 | 10.6 | 17.4 | 2.9 | 10.8 | 0.4 | 0.017±0.039 |
| *Lupinus polyphyllus* | Herb | 4889 | 0.838 | 31.9 | 0.5 | 12.7 | 6 | 16.3 | 0.5 | 0.1 | 25.3 | 6.8 | 0.011±0.011 |
| *Luzula campestris* | Herb | 7129 | 0.799 | 21.5 | 0.9 | 15.3 | 0.9 | 14.1 | 2 | 0 | 27.5 | 17.8 | 0.000±0.000 |
| *Lygodium japonicum* | Vine | 327 | 0.953 | 14.5 | 5 | 14.9 | 1.4 | 0 | 41.8 | 15 | 6.8 | 0.6 | 0.019±0.042 |
| *Lygodium microphyllum* | Fern | 669 | 0.938 | 10.5 | 5.1 | 32.4 | 2.7 | 6.6 | 9.7 | 29.1 | 0.3 | 3.5 | 0.043±0.095 |
| *Lythrum salicaria* | Aquatic Plant | 8902 | 0.765 | 32.3 | 2.1 | 8.5 | 1.3 | 13.4 | 7.2 | 0.3 | 15.8 | 19.1 | 0.009±0.014 |
| *Macfadyena unguis-cati* | Vine | 681 | 0.923 | 16.8 | 1.5 | 50.6 | 0.5 | 0.7 | 13.5 | 5.2 | 10.7 | 0.5 | 0.010±0.012 |
| *Melaleuca quinquenervia* | Tree | 397 | 0.969 | 8.8 | 5.8 | 50.5 | 8.7 | 0.2 | 15.7 | 0.6 | 7 | 2.7 | 0.010±0.012 |
| *Melia azedarach* | Tree | 1192 | 0.89 | 40.4 | 0.2 | 17.8 | 3.7 | 15.2 | 16 | 0.2 | 6.2 | 0.4 | 0.022±0.038 |
| *Melilotus alba* | Herb | 431 | 0.926 | 51.9 | 2.1 | 12.6 | 9.2 | 0.7 | 5 | 2 | 0.5 | 15.9 | 0.009±0.010 |
| *Melinis minutiflora* | Herb | 475 | 0.945 | 11.9 | 0.2 | 51.1 | 4.8 | 4.1 | 22.3 | 0.7 | 2.6 | 2.2 | 0.007±0.008 |
| *Merremia tuberosa* | Vine | 139 | 0.964 | 0.1 | 0.4 | 50.6 | 0.2 | 15.5 | 10.5 | 8.2 | 12.3 | 2.4 | 0.007±0.013 |
| *Miconia calvescens* | Tree | 261 | 0.953 | 3.3 | 0.7 | 62.9 | 5.2 | 8.2 | 6.5 | 0.2 | 11.8 | 1.2 | 0.008±0.009 |
| *Microstegium vimineum* | Herb | 282 | 0.975 | 26 | 0.8 | 15.8 | 4.1 | 0.6 | 33.8 | 6 | 12.9 | 0.1 | 0.034±0.077 |
| *Mikania micrantha* | Vine | 745 | 0.919 | 3 | 0.2 | 52.4 | 1.1 | 2.2 | 26.4 | 9.5 | 5.1 | 0.1 | 0.005±0.012 |
| *Mimosa diplotricha* | Vine | 191 | 0.941 | 9.1 | 1.2 | 26.7 | 2.3 | 9 | 17.9 | 28.2 | 5.4 | 0.3 | 0.007±0.008 |
| *Mimosa pigra* | Shrub | 933 | 0.904 | 2.6 | 1.3 | 56 | 1.1 | 24 | 6.4 | 7.6 | 0.6 | 0.4 | 0.021±0.047 |
| *Mimosa pudica* | Herb | 782 | 0.925 | 4.8 | 3.7 | 22.9 | 2.8 | 15.7 | 30.7 | 15.2 | 4 | 0.2 | 0.008±0.009 |
| *Miscanthus sinensis* | Herb | 400 | 0.954 | 20.2 | 6.3 | 15.9 | 9.9 | 2.7 | 20.2 | 2.2 | 22.1 | 0.5 | 0.010±0.014 |
| *Montia fontana* | Aquatic Plant | 6983 | 0.792 | 33.3 | 2.7 | 26.3 | 1.3 | 4.2 | 2.7 | 1.6 | 25.2 | 2.6 | 0.015±0.011 |
| *Morus alba* | Tree | 912 | 0.904 | 47.3 | 0.7 | 12.6 | 2.7 | 24.6 | 7.2 | 1 | 0.9 | 3 | 0.033±0.075 |
| *Myriophyllum aquaticum* | Aquatic Plant | 833 | 0.939 | 38.5 | 0.1 | 3.4 | 3.8 | 25.2 | 7.1 | 1.8 | 19.7 | 0.3 | 0.010±0.008 |
| *Myriophyllum heterophyllum* | Aquatic Plant | 204 | 0.968 | 19.9 | 1.7 | 5.4 | 3.6 | 15.2 | 17.2 | 1.2 | 26 | 9.7 | 0.010±0.022 |
| *Myriophyllum spicatum* | Aquatic Plant | 4579 | 0.84 | 20.3 | 1.6 | 8.9 | 1.3 | 22.4 | 6.4 | 0.2 | 21.5 | 17.3 | 0.056±0.125 |
| *Najas minor* | Aquatic Plant | 347 | 0.966 | 36.2 | 0.6 | 11.7 | 1.4 | 0 | 11.9 | 4.3 | 13.7 | 20.3 | 0.015±0.034 |
| *Nassella neesiana* | Herb | 410 | 0.957 | 33.5 | 1.5 | 49.1 | 2.4 | 2.7 | 0.2 | 7.2 | 3.2 | 0.1 | 0.022±0.048 |
| *Nassella tenuissima* | Herb | 177 | 0.966 | 32.2 | 1.9 | 40 | 4.1 | 11.2 | 4.1 | 2.8 | 1.9 | 1.6 | 0.023±0.046 |
| *Nephrolepis cordifolia* | Fern | 504 | 0.945 | 16.1 | 0.9 | 3.8 | 17.1 | 23.4 | 20.9 | 2.3 | 14.7 | 0.8 | 0.007±0.009 |
| *Nicotiana glauca* | Shrub | 1552 | 0.902 | 36.8 | 0 | 18.8 | 0 | 29.2 | 13.9 | 0.2 | 1 | 0.2 | 0.025±0.029 |
| *Nymphaea odorata* | Aquatic Plant | 406 | 0.942 | 18.9 | 0.8 | 13.3 | 1.9 | 0.3 | 23.1 | 0.7 | 20.9 | 20 | 0.013±0.008 |
| *Nymphoides peltata* | Aquatic Plant | 1218 | 0.944 | 29.7 | 0.7 | 2.4 | 9.1 | 7.5 | 2.6 | 0.1 | 24.9 | 23.2 | 0.010±0.009 |
| *Ocimum gratissimum* | Herb | 368 | 0.929 | 13.4 | 0.9 | 62.3 | 10 | 0.9 | 4.4 | 6.5 | 0.3 | 1.3 | 0.010±0.011 |
| *Oeceoclades maculata* | Herb | 210 | 0.947 | 2 | 0.8 | 52.6 | 0.1 | 7.5 | 24.9 | 4.1 | 7.5 | 0.4 | 0.025±0.027 |
| *Olea europaea* | Tree | 1806 | 0.908 | 45.8 | 1.6 | 19.5 | 0.6 | 23.4 | 4.2 | 3.7 | 0.8 | 0.3 | 0.020±0.023 |
| *Onopordum acanthium* | Herb | 3422 | 0.873 | 28.5 | 0 | 16.9 | 0.6 | 14.8 | 3.2 | 1.9 | 0.1 | 34.1 | 0.007±0.015 |
| *Opuntia monacantha* | Tree | 141 | 0.957 | 32.1 | 8.1 | 24.3 | 3.2 | 6.5 | 8 | 4.9 | 5.1 | 7.8 | 0.023±0.032 |
| *Opuntia stricta* | Shrub | 681 | 0.954 | 21 | 0.6 | 36.6 | 4.4 | 7.4 | 0.9 | 5.9 | 18.3 | 4.9 | 0.021±0.019 |
| *Oxalis corniculata* | Herb | 5377 | 0.805 | 26.1 | 0.6 | 19.4 | 1.3 | 33 | 11.9 | 0.1 | 7.7 | 0 | 0.010±0.012 |
| *Oxalis latifolia* | Herb | 571 | 0.937 | 23.7 | 0.1 | 26.6 | 18.8 | 19.1 | 6.8 | 1.8 | 2.7 | 0.4 | 0.008±0.009 |
| *Oxalis pes-caprae* | Herb | 1107 | 0.941 | 38.2 | 1 | 12.6 | 0 | 42.5 | 1.8 | 2.8 | 1.2 | 0 | 0.009±0.009 |
| *Oxycaryum cubense* | Aquatic Plant | 214 | 0.938 | 0.1 | 1 | 26 | 9.6 | 24.3 | 0.1 | 31.2 | 6.2 | 1.6 | 0.026±0.017 |
| *Paederia foetida* | Vine | 294 | 0.961 | 11.4 | 8.4 | 15.2 | 5.2 | 0.5 | 25.5 | 29.9 | 2.6 | 1.2 | 0.023±0.029 |
| *Panicum repens* | Herb | 541 | 0.927 | 12.1 | 9.5 | 27.4 | 1.5 | 36.2 | 11 | 1.3 | 0.8 | 0.2 | 0.019±0.024 |
| *Parthenium hysterophorus* | Herb | 798 | 0.92 | 18.9 | 1.3 | 38 | 2.8 | 14.7 | 0.2 | 5.7 | 15.6 | 2.8 | 0.041±0.054 |
| *Paspalum scrobiculatum* | Herb | 1512 | 0.891 | 22.2 | 0.3 | 34.4 | 2 | 7.2 | 4.9 | 27.9 | 0.2 | 0.8 | 0.023±0.031 |
| *Paspalum urvillei* | Herb | 620 | 0.943 | 35.8 | 0.2 | 16.2 | 0.1 | 20.4 | 18.5 | 1.1 | 6.5 | 1.2 | 0.027±0.033 |
| *Paspalum vaginatum* | Herb | 766 | 0.922 | 10.2 | 15.2 | 33.5 | 4.9 | 27.1 | 6.2 | 0.4 | 0.6 | 1.9 | 0.020±0.023 |
| *Passiflora edulis* | Vine | 572 | 0.939 | 6.6 | 0.1 | 35.6 | 14.9 | 8.6 | 23.7 | 0.2 | 9.7 | 0.6 | 0.024±0.029 |
| *Passiflora foetida* | Vine | 2613 | 0.849 | 7.2 | 0.2 | 68.7 | 2.9 | 7.5 | 1.5 | 8.5 | 1.7 | 1.7 | 0.007±0.011 |
| *Passiflora suberosa* | Vine | 776 | 0.928 | 9 | 0.9 | 60.8 | 5.2 | 4.1 | 0.8 | 6.5 | 11.2 | 1.5 | 0.013±0.017 |
| *Passiflora tarminiana* | Vine | 111 | 0.985 | 15.7 | 1 | 34.8 | 34 | 5 | 0 | 1.7 | 6.6 | 1.2 | 0.011±0.014 |
| *Paulownia tomentosa* | Tree | 223 | 0.964 | 41.9 | 1.2 | 9.5 | 1.6 | 8.7 | 4.3 | 0.7 | 31.6 | 0.4 | 0.011±0.012 |
| *Persicaria perfoliata* L | Vine | 185 | 0.976 | 22.5 | 3.8 | 19.6 | 2.9 | 0.6 | 42.7 | 1.7 | 4.5 | 1.5 | 0.037±0.051 |
| *Phalaris arundinacea* | Herb | 10466 | 0.743 | 37.9 | 0.1 | 6.7 | 1.5 | 10.4 | 5.1 | 0.5 | 36.5 | 1.3 | 0.013±0.014 |
| *Phoenix canariensis* | Tree | 186 | 0.965 | 37.4 | 3.6 | 8 | 3.3 | 38 | 0.1 | 5.6 | 1.7 | 2.3 | 0.022±0.026 |
| *Phormium tenax* | Shrub | 374 | 0.975 | 19.8 | 1.4 | 29.1 | 7.8 | 6.2 | 0.5 | 0.2 | 23.1 | 11.8 | 0.036±0.039 |
| *Phragmites australis* | Herb | 12638 | 0.709 | 47.2 | 0.8 | 16.2 | 2 | 0.4 | 3.6 | 0.4 | 16.7 | 12.7 | 0.018±0.018 |
| *Phymatosorus scolopendria* | Herb | 511 | 0.947 | 0.8 | 11.4 | 28.7 | 8.3 | 23.6 | 23.2 | 0.1 | 3.7 | 0.2 | 0.018±0.020 |
| *Physalis peruviana* | Shrub | 953 | 0.931 | 4.9 | 0.5 | 13.5 | 36.6 | 26.7 | 12.4 | 2.5 | 2.7 | 0.1 | 0.004±0.006 |
| *Pimenta dioica* | Tree | 130 | 0.958 | 2.4 | 4 | 36.8 | 0 | 20.9 | 5.2 | 6.3 | 18.4 | 5.9 | 0.015±0.008 |
| *Pinus caribaea* | Tree | 141 | 0.97 | 2.2 | 7.2 | 45.5 | 1.4 | 2.2 | 18.7 | 2.8 | 16.3 | 3.6 | 0.023±0.051 |
| *Pinus nigra* | Tree | 3135 | 0.884 | 31.2 | 1.4 | 19.7 | 0.3 | 18 | 6.2 | 1 | 0 | 22.2 | 0.014±0.014 |
| *Pinus pinaster* | Tree | 1549 | 0.932 | 41.2 | 7.7 | 29.6 | 0 | 17.4 | 1.6 | 1.2 | 0.4 | 0.9 | 0.007±0.015 |
| *Piper aduncum* | Tree | 1007 | 0.922 | 1.8 | 0.8 | 68.5 | 1.9 | 1.3 | 15.8 | 1.2 | 8.7 | 0 | 0.018±0.023 |
| *Pistia stratiotes* | Aquatic Plant | 655 | 0.891 | 13.7 | 3 | 21.8 | 2.2 | 19.1 | 34.7 | 0.1 | 4.7 | 0.6 | 0.029±0.034 |
| *Pittosporum tenuifolium* | Tree | 281 | 0.982 | 18.5 | 1.4 | 32.2 | 8.2 | 7.5 | 0.5 | 0.1 | 22.5 | 9.1 | 0.038±0.048 |
| *Pittosporum undulatum* | Tree | 601 | 0.967 | 33.6 | 1 | 32.7 | 7.3 | 4.7 | 0 | 2.7 | 17.8 | 0.3 | 0.014±0.016 |
| *Pittosporum viridiflorum* | Tree | 304 | 0.965 | 20.1 | 1.1 | 43.3 | 24.2 | 0.1 | 5 | 0.7 | 5.1 | 0.4 | 0.016±0.035 |
| *Pluchea carolinensis* | Shrub | 414 | 0.952 | 5.1 | 0.8 | 58.3 | 2.1 | 12.8 | 0.4 | 5.2 | 11.1 | 4.3 | 0.027±0.031 |
| *Pluchea indica* | Shrub | 130 | 0.979 | 5.9 | 23.3 | 9.9 | 0.8 | 49 | 7.4 | 0.5 | 0.2 | 3 | 0.006±0.013 |
| *Poa annua* | Herb | 13844 | 0.705 | 39.6 | 1.9 | 31.6 | 0.3 | 12.5 | 8.2 | 0.6 | 5 | 0.4 | 0.015±0.021 |
| *Poa pratensis* | Herb | 15673 | 0.685 | 56.1 | 0 | 26.9 | 2.3 | 4.8 | 0 | 0.3 | 6.5 | 3 | 0.007±0.008 |
| *Polygala paniculata* | Herb | 636 | 0.944 | 1.6 | 0.7 | 37.1 | 12.4 | 10.8 | 27.2 | 2.5 | 6.2 | 1.5 | 0.009±0.009 |
| *Polygonum cuspidatum* | Herb | 240 | 0.959 | 29.5 | 2 | 10.3 | 2.7 | 4.9 | 37.1 | 0.3 | 11.3 | 2 | 0.021±0.026 |
| *Polysiphonia brodiei* | Alga | 227 | 0.985 | 11.7 | 26.7 | 8.7 | 16.2 | 11.9 | 0.2 | 1.2 | 14 | 9.5 | 0.013±0.012 |
| *Populus alba* | Tree | 4048 | 0.855 | 40.8 | 2.7 | 12.2 | 0.4 | 19.3 | 6.2 | 1.8 | 0.4 | 16.3 | 0.011±0.026 |
| *Potamogeton crispus* | Aquatic Plant | 4017 | 0.847 | 29 | 1.1 | 8.8 | 0.7 | 24 | 5.8 | 0 | 21.2 | 9.3 | 0.010±0.023 |
| *Potamogeton perfoliatus* | Aquatic Plant | 4880 | 0.832 | 9.7 | 1.4 | 13.4 | 12.5 | 4.7 | 0.1 | 1.3 | 41.7 | 15.1 | 0.010±0.008 |
| *Prosopis glandulosa* | Tree | 574 | 0.947 | 17.9 | 31.5 | 9.2 | 6.6 | 13.8 | 11.8 | 2.7 | 0.8 | 5.8 | 0.023±0.028 |
| *Prosopis juliflora* | Shrub | 282 | 0.915 | 31.9 | 1.2 | 28.7 | 2.6 | 16 | 13.3 | 1.1 | 2.2 | 3.1 | 0.007±0.009 |
| *Psidium cattleianum* | Tree | 111 | 0.959 | 7.9 | 4.6 | 18.5 | 14.3 | 29.5 | 6.8 | 1 | 16.2 | 1.1 | 0.009±0.014 |
| *Psidium guajava* | Tree | 972 | 0.907 | 8 | 0.2 | 37.7 | 0.6 | 17 | 8.9 | 18.9 | 8.3 | 0.4 | 0.011±0.012 |
| *Psoralea pinnata* | Shrub | 221 | 0.977 | 28.2 | 0.1 | 42.1 | 7.3 | 7 | 1.2 | 2.7 | 8.9 | 2.4 | 0.017±0.038 |
| *Pteris cretica* | Fern | 520 | 0.947 | 33.3 | 1.1 | 11 | 13.5 | 14.7 | 20.5 | 2.8 | 1 | 2.1 | 0.006±0.010 |
| *Pyrus calleryana* | Tree | 118 | 0.952 | 39.4 | 1 | 16.7 | 2 | 7.9 | 27.1 | 1 | 3.3 | 1.6 | 0.051±0.070 |
| *Ranunculus ficaria* | Herb | 7226 | 0.797 | 22.3 | 6.8 | 12 | 0.2 | 22.2 | 5.8 | 0.3 | 11 | 19.6 | 0.014±0.016 |
| *Rauvolfia vomitoria* | Tree | 416 | 0.967 | 0.2 | 3.4 | 63.6 | 0.3 | 11.3 | 13.5 | 2.9 | 2.9 | 1.8 | 0.023±0.052 |
| *Rhamnus alaternus* | Tree | 1142 | 0.946 | 29.3 | 10 | 28.8 | 2.3 | 22 | 3.6 | 3.7 | 0.1 | 0.2 | 0.012±0.027 |
| *Rhamnus cathartica* | Tree | 4063 | 0.86 | 33.5 | 0.2 | 5.7 | 3.4 | 3.3 | 1.7 | 2.1 | 21.5 | 28.6 | 0.018±0.022 |
| *Rhizophora mangle* | Aquatic Plant | 480 | 0.944 | 5.3 | 2.1 | 29.5 | 7 | 53.3 | 0.8 | 0.6 | 1.1 | 0.4 | 0.036±0.039 |
| *Rhododendron ponticum* | Shrub | 1737 | 0.931 | 12.8 | 19.1 | 14.3 | 7.8 | 6.3 | 0.4 | 0.6 | 27.1 | 11.6 | 0.022±0.027 |
| *Rhus longipes* | Tree | 103 | 0.969 | 9.9 | 2.6 | 62.5 | 0.7 | 4.6 | 9.4 | 7.3 | 1.1 | 1.8 | 0.013±0.028 |
| *Ricinus communis* | Tree | 1423 | 0.86 | 13.5 | 1.1 | 29.5 | 7.2 | 37.5 | 7.5 | 2 | 1.7 | 0.1 | 0.016±0.022 |
| *Robinia pseudoacacia* | Tree | 4794 | 0.837 | 49.2 | 0 | 5.2 | 0.5 | 16.8 | 6 | 0.3 | 15.4 | 6.7 | 0.009±0.009 |
| *Rosa multiflora* | Shrub | 1009 | 0.936 | 44.4 | 1.1 | 2.6 | 0.9 | 13.1 | 9.1 | 0.2 | 28.3 | 0.2 | 0.007±0.015 |
| *Rottboellia cochinchinensis* | Herb | 506 | 0.908 | 28.4 | 1.9 | 27.9 | 0.9 | 3.1 | 1.2 | 34.6 | 0.8 | 1.2 | 0.005±0.011 |
| *Rubus discolor* | Vine | 864 | 0.956 | 38.2 | 0 | 15.1 | 1.3 | 18.5 | 0.5 | 0.9 | 8.2 | 17.3 | 0.005±0.007 |
| *Rubus moluccanus* | Vine | 525 | 0.964 | 8.9 | 0.5 | 35.3 | 12.6 | 5.3 | 8.9 | 0.1 | 28.1 | 0.3 | 0.020±0.027 |
| *Rubus niveus* | Shrub | 167 | 0.962 | 15 | 4.9 | 6.2 | 18.1 | 9.4 | 26.5 | 4.9 | 3.3 | 11.7 | 0.026±0.034 |
| *Rubus phoenicolasius* | Shrub | 232 | 0.98 | 44.9 | 0.1 | 2.9 | 2.4 | 1.4 | 3.3 | 0.1 | 44 | 1 | 0.025±0.029 |
| *Rubus pinnatus* | Shrub | 144 | 0.98 | 20 | 0.7 | 49 | 15.8 | 0 | 3.8 | 1 | 7.8 | 1.8 | 0.032±0.042 |
| *Rubus rosifolius* | Tree | 600 | 0.96 | 16.2 | 0.2 | 11.5 | 19.7 | 12.2 | 18.2 | 0.4 | 21.5 | 0.1 | 0.019±0.028 |
| *Rubus ulmifolius* | Vine | 3778 | 0.865 | 39.4 | 1.3 | 38.3 | 0.6 | 16.2 | 2.5 | 0.6 | 0.2 | 0.9 | 0.009±0.010 |
| *Ruellia brevifolia* | Herb | 151 | 0.952 | 12.3 | 2.5 | 49.8 | 5.7 | 5.9 | 7.6 | 4.3 | 11 | 0.9 | 0.020±0.029 |
| *Rumex acetosella* | Herb | 13661 | 0.708 | 36.2 | 0.8 | 21.1 | 0.3 | 12.6 | 16.3 | 0.3 | 11.2 | 1.3 | 0.020±0.046 |
| *Rumex crispus* | Herb | 10230 | 0.744 | 40.9 | 0.2 | 13.7 | 1 | 34.6 | 5.4 | 0.4 | 0 | 3.8 | 0.013±0.029 |
| *Rumex obtusifolius* | Herb | 7627 | 0.788 | 22 | 0.2 | 4.7 | 4.8 | 25.9 | 1.3 | 0.5 | 29.4 | 11.2 | 0.037±0.036 |
| *Sacciolepis indica* | Herb | 764 | 0.936 | 17 | 2.2 | 6.7 | 5.4 | 1.4 | 27.8 | 32.9 | 2.8 | 3.8 | 0.010±0.013 |
| *Sagina procumbens* | Herb | 10122 | 0.752 | 13.1 | 2.1 | 11.6 | 12.7 | 7.5 | 0.5 | 0 | 45.1 | 7.3 | 0.017±0.022 |
| *Sagittaria platyphylla* | Aquatic Plant | 158 | 0.976 | 46.4 | 4 | 3.6 | 1.9 | 7.8 | 3.9 | 1.2 | 29.2 | 1.9 | 0.016±0.015 |
| *Sagittaria sagittifolia* | Aquatic Plant | 3205 | 0.881 | 2.4 | 11.2 | 6.5 | 21.8 | 10 | 0.2 | 1.1 | 21.2 | 25.6 | 0.019±0.007 |
| *Salix babylonica* | Tree | 852 | 0.936 | 44.6 | 0.1 | 11 | 1.3 | 23.6 | 4.4 | 0.6 | 13.5 | 1 | 0.016±0.017 |
| *Salix cinerea* | Tree | 7615 | 0.79 | 6.8 | 1.2 | 4.7 | 15.6 | 13.2 | 0.2 | 0.3 | 39.4 | 18.7 | 0.018±0.024 |
| *Salix humboldtiana* | Tree | 325 | 0.943 | 3.1 | 6.6 | 62.1 | 2 | 4 | 8.8 | 0.1 | 9.7 | 3.7 | 0.026±0.025 |
| *Salsola tragus* | Shrub | 3200 | 0.852 | 11.9 | 23.9 | 21.6 | 3.2 | 12.5 | 17.4 | 0.8 | 6.9 | 1.9 | 0.008±0.013 |
| *Salvinia minima* | Aquatic Plant | 167 | 0.944 | 17.3 | 4.3 | 15.1 | 11.3 | 2.3 | 29.3 | 8.8 | 10.1 | 1.5 | 0.022±0.029 |
| *Salvinia molesta* | Aquatic Plant | 273 | 0.936 | 27.4 | 0.4 | 23.9 | 2.5 | 21.1 | 9.4 | 1.2 | 11 | 3.1 | 0.015±0.018 |
| *Samanea saman* | Tree | 185 | 0.945 | 0.5 | 5.1 | 30 | 1.8 | 31.9 | 7.8 | 19.2 | 0.9 | 2.8 | 0.017±0.022 |
| *Sansevieria hyacinthoides* | Succulent | 124 | 0.966 | 9.3 | 0.6 | 54.3 | 6.4 | 0.3 | 3 | 8.8 | 14.8 | 2.6 | 0.012±0.014 |
| *Sargassum muticum* | Aquatic Plant | 348 | 0.98 | 26.3 | 28.9 | 4.8 | 13.4 | 17 | 0.1 | 0.1 | 8.9 | 0.7 | 0.020±0.025 |
| *Scaevola sericea* | Shrub | 150 | 0.97 | 1.8 | 30.6 | 5.1 | 3.1 | 46.6 | 11.3 | 0.2 | 0.8 | 0.5 | 0.025±0.031 |
| *Schefflera actinophylla* | Tree | 240 | 0.969 | 14.4 | 12 | 46.6 | 2.5 | 0.3 | 17.6 | 0.2 | 2.9 | 3.4 | 0.028±0.036 |
| *Schinus terebinthifolius* | Tree | 306 | 0.957 | 20.5 | 0.9 | 41.4 | 4 | 19.5 | 0.2 | 2.9 | 6.3 | 4.4 | 0.022±0.049 |
| *Schismus arabicus* | Herb | 440 | 0.953 | 19.1 | 3 | 22.6 | 1.9 | 14.3 | 21 | 2.8 | 14.2 | 1 | 0.007±0.007 |
| *Schismus barbatus* | Herb | 1870 | 0.912 | 27.3 | 0.1 | 6.8 | 0.4 | 33.9 | 12.7 | 16.1 | 0.2 | 2.4 | 0.010±0.012 |
| *Sechium edule* | Vine | 134 | 0.946 | 17.4 | 0.2 | 17.8 | 13.1 | 6.6 | 10.3 | 27 | 5.5 | 2.1 | 0.022±0.011 |
| *Senecio angulatus* | Herb | 156 | 0.986 | 34.1 | 8.5 | 17.6 | 5.1 | 25.8 | 2.9 | 5.6 | 0.1 | 0.3 | 0.023±0.051 |
| *Senecio inaequidens* | Shrub | 1550 | 0.928 | 21.3 | 0.1 | 31.5 | 11.3 | 11.8 | 7 | 3.3 | 0.3 | 13.3 | 0.009±0.021 |
| *Senecio jacobaea* | Herb | 6593 | 0.808 | 22 | 6.1 | 14 | 1.2 | 19.1 | 1.3 | 0.1 | 21.4 | 14.9 | 0.014±0.018 |
| *Senecio squalidus* | Herb | 1155 | 0.948 | 22 | 9.8 | 26.4 | 0.8 | 9.4 | 0.8 | 2.8 | 17.2 | 10.9 | 0.010±0.010 |
| *Senecio viscosus* | Herb | 5371 | 0.831 | 5.1 | 5.1 | 3.1 | 16.8 | 16.3 | 0.2 | 0 | 33.2 | 20.1 | 0.026±0.058 |
| *Senecio vulgaris* | Herb | 9706 | 0.755 | 40.5 | 3.1 | 24.7 | 0.6 | 17.3 | 5.1 | 1.1 | 2.1 | 5.6 | 0.042±0.093 |
| *Sesbania punicea* | Shrub | 135 | 0.966 | 45.3 | 0.7 | 24.4 | 2.2 | 1.1 | 3.7 | 15.6 | 5.1 | 1.8 | 0.015±0.023 |
| *Setaria verticillata* | Herb | 2903 | 0.861 | 19.6 | 2.6 | 25.8 | 1.9 | 30.5 | 8.8 | 10.6 | 0.1 | 0.1 | 0.009±0.013 |
| *Solanum mauritianum* | Tree | 474 | 0.965 | 13.8 | 0.3 | 37.1 | 22.8 | 14.8 | 0.5 | 2.2 | 7.1 | 1.3 | 0.023±0.028 |
| *Solanum seaforthianum* | Vine | 482 | 0.942 | 24.8 | 1.1 | 41.1 | 5 | 0.1 | 10.1 | 8.2 | 8 | 1.6 | 0.034±0.035 |
| *Solanum sisymbriifolium* | Herb | 289 | 0.938 | 13.3 | 0.3 | 23.1 | 3.6 | 16.7 | 31.3 | 5.8 | 2 | 3.8 | 0.008±0.014 |
| *Solanum torvum* | Shrub | 1050 | 0.919 | 1.6 | 2.9 | 29.4 | 3.6 | 9.2 | 15.7 | 34.4 | 2.1 | 1.1 | 0.010±0.012 |
| *Solidago canadensis* | Herb | 4903 | 0.829 | 45.2 | 0.2 | 7.5 | 3.4 | 0.1 | 3.4 | 0.9 | 24.3 | 15.1 | 0.007±0.015 |
| *Sorghum halepense* | Herb | 2477 | 0.847 | 25.4 | 0.4 | 13.2 | 5.1 | 43.8 | 7.3 | 2.9 | 1.8 | 0.1 | 0.013±0.018 |
| *Spartina alterniflora* | Herb | 199 | 0.97 | 13.8 | 6.3 | 8.2 | 6.8 | 1.4 | 7.2 | 1.9 | 49.4 | 5.1 | 0.015±0.029 |
| *Spartina anglica* | Herb | 380 | 0.981 | 18.8 | 2.7 | 15.6 | 29.8 | 0.5 | 0.2 | 1 | 20 | 11.4 | 0.011±0.013 |
| *Spartium junceum* | Shrub | 988 | 0.945 | 43.3 | 2.3 | 24.1 | 0 | 22.9 | 2.6 | 3.4 | 1.3 | 0 | 0.005±0.006 |
| *Spathodea campanulata* | Tree | 247 | 0.939 | 3.5 | 7.9 | 49.1 | 4.8 | 10.8 | 9.2 | 12.6 | 1.5 | 0.6 | 0.020±0.023 |
| *Spermacoce verticillata* | Shrub | 685 | 0.922 | 0.8 | 0.7 | 64.2 | 1.4 | 6.3 | 0.1 | 16.3 | 10.2 | 0.1 | 0.006±0.009 |
| *Sphagneticola trilobata* | Herb | 469 | 0.948 | 1.8 | 8.1 | 22.5 | 2.5 | 30.6 | 22.8 | 3.4 | 7.9 | 0.3 | 0.013±0.030 |
| *Spiraea japonica* | Shrub | 535 | 0.953 | 28.2 | 2.4 | 22.6 | 5.7 | 14.8 | 12.2 | 0.7 | 12.9 | 0.5 | 0.010±0.011 |
| *Stellaria alsine* | Herb | 6475 | 0.808 | 14.6 | 6.7 | 16.5 | 4.4 | 13.9 | 0.4 | 0 | 42.2 | 1.3 | 0.032±0.038 |
| *Stellaria media* | Herb | 12988 | 0.713 | 37.6 | 1.1 | 30.5 | 0.6 | 10.1 | 10.5 | 0.4 | 7.2 | 2 | 0.013±0.020 |
| *Striga asiatica* | Herb | 292 | 0.925 | 20 | 0.8 | 32.8 | 2.7 | 5.1 | 9.8 | 24.2 | 1 | 3.6 | 0.039±0.088 |
| *Syngonium podophyllum* | Vine | 584 | 0.947 | 3.6 | 0.5 | 42.2 | 0 | 10.7 | 26.5 | 1.8 | 14.4 | 0.2 | 0.011±0.013 |
| *Syzygium cumini* | Tree | 155 | 0.932 | 1.2 | 10.4 | 7.7 | 0.9 | 32.9 | 9 | 34.7 | 2.3 | 0.9 | 0.028±0.031 |
| *Syzygium jambos* | Tree | 448 | 0.942 | 4.7 | 0.7 | 22.6 | 6.2 | 14.1 | 29.3 | 12.8 | 8.1 | 1.6 | 0.025±0.031 |
| *Tamarix aphylla* | Shrub | 267 | 0.939 | 32.3 | 1.5 | 3.5 | 0.7 | 31.2 | 26.4 | 1.1 | 0.9 | 2.4 | 0.013±0.015 |
| *Tamarix parviflora* | Tree | 292 | 0.959 | 44.4 | 0.2 | 9.3 | 1 | 27.8 | 8 | 0.5 | 5.8 | 2.9 | 0.010±0.010 |
| *Tamarix ramosissima* | Tree | 765 | 0.93 | 37.5 | 14.1 | 7.8 | 5.3 | 13.8 | 5.8 | 10.3 | 3 | 2.5 | 0.022±0.042 |
| *Taraxacum officinale* | Herb | 4946 | 0.811 | 39.8 | 0.3 | 16.2 | 0.5 | 22.2 | 9.1 | 0.3 | 10.8 | 0.7 | 0.036±0.046 |
| *Tecoma capensis* | Vine | 219 | 0.959 | 21.1 | 4.4 | 28.3 | 9.6 | 26.3 | 4.2 | 3.8 | 1 | 1.3 | 0.038±0.048 |
| *Tecoma stans* | Tree | 630 | 0.915 | 29.3 | 2.4 | 44.5 | 2.2 | 0.3 | 3.9 | 4.8 | 9 | 3.6 | 0.024±0.029 |
| *Terminalia catappa* | Tree | 374 | 0.945 | 1.9 | 10.5 | 9.7 | 1.1 | 57.1 | 17.7 | 0.5 | 1 | 0.6 | 0.021±0.019 |
| *Thevetia peruviana* | Tree | 353 | 0.923 | 18.1 | 1.5 | 37.3 | 5.3 | 19.4 | 9.1 | 0.5 | 6.4 | 2.4 | 0.029±0.040 |
| *Thunbergia grandiflora* | Vine | 153 | 0.939 | 3.3 | 6.5 | 24.8 | 5 | 13 | 39.2 | 4.3 | 3.2 | 0.7 | 0.035±0.052 |
| *Tithonia diversifolia* | Shrub | 380 | 0.945 | 6.2 | 0.7 | 31.8 | 2.3 | 13.1 | 16.7 | 19.4 | 6.6 | 3.2 | 0.024±0.037 |
| *Tradescantia fluminensis* | Herb | 476 | 0.963 | 28.6 | 0.2 | 11.5 | 10.6 | 22.6 | 4.5 | 2 | 17 | 3.1 | 0.007±0.016 |
| *Trapa natans* | Aquatic Plant | 340 | 0.954 | 28.5 | 7.2 | 12 | 5.2 | 1.2 | 12.5 | 0.3 | 22.1 | 10.9 | 0.029±0.037 |
| *Triadica sebifera* | Tree | 200 | 0.967 | 33 | 1.3 | 18.5 | 0 | 4.1 | 19.6 | 11.7 | 10.8 | 1 | 0.023±0.027 |
| *Trifolium dubium* | Herb | 6214 | 0.811 | 29.6 | 0.3 | 13.5 | 0.5 | 26.9 | 7.8 | 0.1 | 12.9 | 8.4 | 0.021±0.024 |
| *Trifolium repens* | Herb | 14055 | 0.703 | 40 | 0.2 | 23.6 | 0.7 | 10.4 | 8.9 | 0.5 | 14.3 | 1.4 | 0.013±0.014 |
| *Tussilago farfara* | Herb | 8694 | 0.77 | 13.4 | 1.1 | 8.7 | 9.1 | 7.9 | 0.1 | 0.2 | 40.3 | 19.1 | 0.013±0.014 |
| *Typha latifolia* | Aquatic Plant | 7085 | 0.786 | 50.2 | 0 | 6.2 | 3.8 | 8.4 | 6 | 1.5 | 15.1 | 8.8 | 0.011±0.013 |
| *Ulex europaeus* | Tree | 3828 | 0.867 | 26 | 1.2 | 26.3 | 5.5 | 14.9 | 1 | 0.3 | 16.7 | 8.1 | 0.038±0.086 |
| *Urochloa mutica* | Herb | 309 | 0.927 | 7.6 | 3.1 | 46.5 | 2.7 | 18.5 | 1.7 | 14.9 | 2.5 | 2.5 | 0.042±0.094 |
| *Utricularia gibba* | Aquatic Plant | 790 | 0.9 | 24.8 | 0.9 | 9.3 | 5 | 0.7 | 22.3 | 32.8 | 2.1 | 2.1 | 0.016±0.035 |
| *Vallisneria nana* | Aquatic Plant | 220 | 0.957 | 8.5 | 6.1 | 48.6 | 7.1 | 10.8 | 10.8 | 0.4 | 6.4 | 1.3 | 0.009±0.020 |
| *Vallisneria spiralis* | Aquatic Plant | 242 | 0.95 | 11 | 1.9 | 24.9 | 7.4 | 6.2 | 11.5 | 0.2 | 0.7 | 36.1 | 0.016±0.017 |
| *Verbascum thapsus* | Herb | 6147 | 0.805 | 32.1 | 0 | 9 | 0.7 | 26.7 | 5.3 | 0.8 | 10.7 | 14.7 | 0.011±0.021 |
| *Verbena brasiliensis* | Herb | 188 | 0.976 | 44.8 | 0.8 | 8.1 | 0.8 | 14.6 | 3.6 | 18.9 | 0.9 | 7.4 | 0.027±0.025 |
| *Verbena rigida* | Herb | 562 | 0.961 | 22.1 | 1.1 | 25.2 | 4.8 | 11.2 | 15.2 | 3.4 | 15.6 | 1.4 | 0.011±0.020 |
| *Vinca major* | Herb | 2468 | 0.899 | 38.7 | 0.6 | 15.6 | 5.7 | 24.6 | 4.5 | 0.5 | 7.4 | 2.5 | 0.009±0.009 |
| *Vitex rotundifolia* | Shrub | 215 | 0.979 | 6.6 | 22.1 | 18 | 9.5 | 2.3 | 7.4 | 31 | 1.2 | 2 | 0.011±0.024 |
| *Vulpia bromoides* | Herb | 4787 | 0.838 | 42.7 | 0 | 27.3 | 1.1 | 26.1 | 0.8 | 0.3 | 0.5 | 1.3 | 0.023±0.025 |
| *Waterhousea floribunda* | Tree | 130 | 0.992 | 9.1 | 1.1 | 47.7 | 10.7 | 5.1 | 6.6 | 0.3 | 19.3 | 0.1 | 0.011±0.010 |
| *Wisteria floribunda* | Vine | 144 | 0.98 | 29.5 | 3.5 | 14.1 | 0.9 | 5.3 | 16.9 | 3.8 | 25.2 | 0.8 | 0.006±0.008 |
| *Wisteria sinensis* | Vine | 160 | 0.964 | 30.4 | 1.8 | 12 | 2.3 | 18.9 | 4.8 | 1 | 16.7 | 12.2 | 0.014±0.018 |
| *Xanthium spinosum* | Herb | 1935 | 0.899 | 30.6 | 0.6 | 23.2 | 2.1 | 29.5 | 5.7 | 7 | 0.4 | 0.9 | 0.009±0.020 |
| *Zantedeschia aethiopica* | Herb | 473 | 0.964 | 37.4 | 1.5 | 35.9 | 4.3 | 13.8 | 3.1 | 2.7 | 0.3 | 1 | 0.014±0.007 |
| *Zizania latifolia* | Herb | 116 | 0.985 | 22.2 | 2.8 | 18.3 | 11.1 | 0.5 | 26.3 | 13.9 | 4.5 | 0.5 | 0.008±0.008 |
| *Ziziphus mauritiana* | Tree | 283 | 0.924 | 33.2 | 4 | 25.3 | 0.9 | 11.3 | 3.2 | 17.4 | 0.7 | 4 | 0.011±0.014 |
| Mean |  | 1915.023 | 0.915 | 22.52 | 3.117 | 23.928 | 5.128 | 15.331 | 9.592 | 5.112 | 10.632 | 4.638 |  |
