# Supplementary material for: Potential invasive plant expansion in global ecoregions under climate change
Source: PeerJ. 2019 Mar 5;7:e6479. doi: 10.7717/peerj.6479 (PMC6407507; doi:10.7717/peerj.6479)
Supplement: Supplemental Information 2 — Terrestrial represents terrestrial ecoregions; Freshwater represents freshwater ecoregions; Codes used in this table are defined as follows: For terrestrial ecoregions: BF: Boreal Forests/Taiga; DXS: Deserts and Xeric Shrublands; FGS: Flooded Grasslands and Savannas; IW: Inland Water; MG: Mangroves; MFWS: Mediterranean Forests, Woodlands and Scrub; MGS: Montane Grasslands and Shrublands; RI: Rock and Ice; TBMF: Temperate Broadleaf and Mixed Forests; TCF: Temperate Conifer Forests; TGSS: Temperate Grasslands, Savannas and Shrublands; TSCF: Tropical and Subtropical Coniferous Forests; TSDBF: Tropical and Subtropical Dry Broadleaf Forests; TSGSS: Tropical and Subtropical Grasslands, Savannas and Shrublands; TSMBF: Tropical and Subtropical Moist Broadleaf Forests; TD: Tundra. For freshwater ecoregions: LL: Large Lakes; LRD: Large River Deltas; MF: Montane Freshwaters; OI: Oceanic Islands; PF: Polar Freshwaters; TCR: Temperate Coastal Rivers; TFRW: Temperate Floodplain Rivers and Wetlands; TUR: Temperate Upland Rivers; TSCR: Tropical and Subtropical Coastal Rivers; TSFRWC: Tropical and Subtropical Floodplain Rivers and Wetland Complexes; TSUR: Tropical and Subtropical Upland Rivers; XFEB: Xeric Freshwaters and Endorheic (closed) Basins. P < 0.05*; P < 0.01**; P < 0.001***. [file peerj-07-6479-s002.docx]

**Table S2. The relationships of the expansion potential of IPS between RCPs 4.5 and 8.5 scenarios based on logistic values of Maxent.**

| Terrestrial | | |  | Freshwater | | |
| --- | --- | --- | --- | --- | --- | --- |
| Code | R^2^ | *F* value |  | Code | R^2^ | *F* value |
| BF | 0.9683^***^ | 611.7429 |  | LL | 0.9771^***^ | 510.9380 |
| DXS | 0.6993^***^ | 216.2381 |  | LRD | 0.7509^ns^ | 6.0282 |
| FGS | 0.4107^***^ | 16.7237 |  | MF | 0.9102^***^ | 415.7224 |
| IW | 0.9987^*^ | 741.0307 |  | OI | 0.8035^*^ | 159.4343 |
| MG | 0.4997^***^ | 16.9824 |  | PF | 0.9345^***^ | 256.7990 |
| MFWS | 0.4734^***^ | 33.2616 |  | TCR | 0.8987^***^ | 470.2548 |
| MGS | 0.8872^***^ | 377.6961 |  | TFRW | 0.6538^***^ | 43.4290 |
| RI | 0.9999^***^ | 15738.4900 |  | TUR | 0.7287^***^ | 51.0249 |
| TBMF | 0.9093^***^ | 861.7966 |  | TSCR | 0.4516^***^ | 70.0010 |
| TCF | 0.9537^***^ | 948.1391 |  | TSFRWC | 0.3245^***^ | 22.5797 |
| TGSS | 0.9775^***^ | 1648.7290 |  | TSUR | 0.8275^***^ | 129.5463 |
| TSCF | 0.7298^***^ | 40.5244 |  | XFEB | 0.565^***^ | 75.3200 |
| TSDBF | 0.8157^***^ | 234.6318 |  | Palm | 0.8897^***^ | 3597.8670 |
| TSGSS | 0.7575^***^ | 143.6680 |  | Herb | 0.8214^***^ | 2051.8880 |
| TSMBF | 0.3734^***^ | 135.8462 |  | Tree | 0.7499^***^ | 1337.0190 |
| TD | 0.9791^***^ | 1308.6810 |  | Shrub | 0.7468^***^ | 1315.2940 |
| Palm | 0.8781^***^ | 5848.7330 |  | Vine | 0.8278^***^ | 2144.0670 |
| Herb | 0.8624^***^ | 5089.7170 |  | Aquatic | 0.7636^***^ | 1440.3850 |
| Tree | 0.8039^***^ | 3329.2420 |  | Fern | 0.7794^***^ | 1575.5100 |
| Shrub | 0.8325^***^ | 4034.6530 |  | Alga | 0.7891^***^ | 1669.1860 |
| Vine | 0.8262^***^ | 3860.0490 |  | Succulent | 0.5959^***^ | 657.6794 |
| Aquatic | 0.7852^***^ | 2968.4460 |  | All biomes | 0.8108^***^ | 1910.8020 |
| Fern | 0.7960^***^ | 3167.4760 |  |  |  |  |
| Alga | 0.7742^***^ | 2784.1780 |  |  |  |  |
| Succulent | 0.6821^***^ | 1742.5340 |  |  |  |  |
| All biomes | 0.8478^***^ | 4522.3950 |  |  |  |  |

Terrestrial represents terrestrial ecoregions; Freshwater represents freshwater ecoregions; Codes used in this table are defined as follows: For terrestrial ecoregions: BF: Boreal Forests/Taiga; DXS: Deserts and Xeric Shrublands; FGS: Flooded Grasslands and Savannas; IW: Inland Water; MG: Mangroves; MFWS: Mediterranean Forests, Woodlands and Scrub; MGS: Montane Grasslands and Shrublands; RI: Rock and Ice; TBMF: Temperate Broadleaf and Mixed Forests; TCF: Temperate Conifer Forests; TGSS: Temperate Grasslands, Savannas and Shrublands; TSCF: Tropical and Subtropical Coniferous Forests; TSDBF: Tropical and Subtropical Dry Broadleaf Forests; TSGSS: Tropical and Subtropical Grasslands, Savannas and Shrublands; TSMBF: Tropical and Subtropical Moist Broadleaf Forests; TD: Tundra. For freshwater ecoregions: LL: Large Lakes; LRD: Large River Deltas; MF: Montane Freshwaters; OI: Oceanic Islands; PF: Polar Freshwaters; TCR: Temperate Coastal Rivers; TFRW: Temperate Floodplain Rivers and Wetlands; TUR: Temperate Upland Rivers; TSCR: Tropical and Subtropical Coastal Rivers; TSFRWC: Tropical and Subtropical Floodplain Rivers and Wetland Complexes; TSUR: Tropical and Subtropical Upland Rivers; XFEB: Xeric Freshwaters and Endorheic (closed) Basins. *P*<0.05^*^; *P*<0.01^**^; *P*<0.001^***^.
