# Supplementary figures and images for: Potential invasive plant expansion in global ecoregions under climate change
Source: PeerJ. 2019 Mar 5;7:e6479. doi: 10.7717/peerj.6479 (PMC6407507; doi:10.7717/peerj.6479)

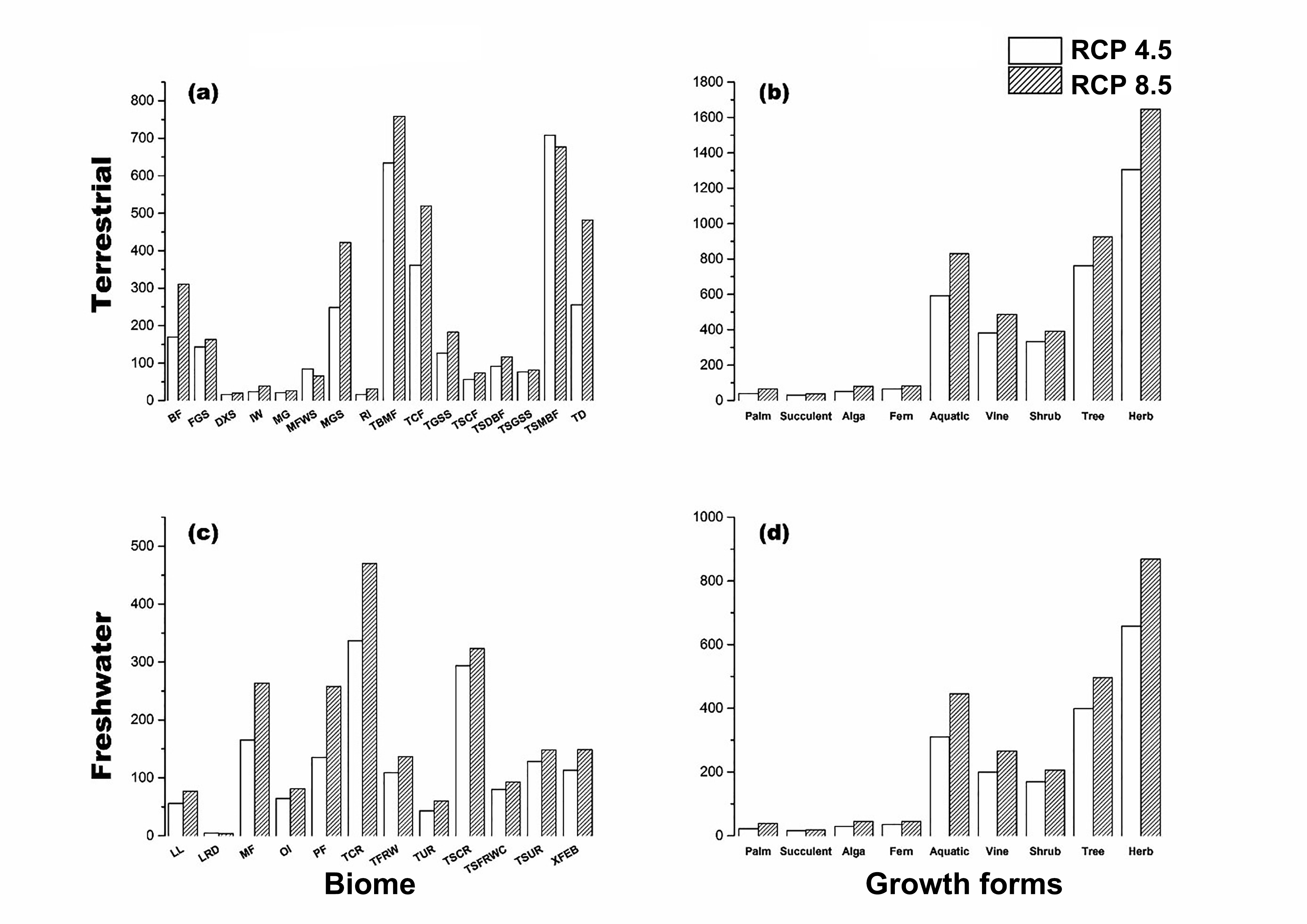

Supplement: Supplemental Information 3 — The color coupling with the numbers of this figure represents the level of IPS expansion potential across different ecoregions. The numbers of this figure represent the degrees of potential of invasive plant expansion. Terrestrial represents terrestrial ecoregions; Freshwater represents freshwater ecoregions; Codes used in this figure are defined as follows: For terrestrial ecoregions: BF: Boreal Forests/Taiga; DXS: Deserts and Xeric Shrublands; FGS: Flooded Grasslands and Savannas; IW: Inland Water; MG: Mangroves; MFWS: Mediterranean Forests, Woodlands and Scrub; MGS: Montane Grasslands and Shrublands; RI: Rock and Ice; TBMF: Temperate Broadleaf and Mixed Forests; TCF: Temperate Conifer Forests; TGSS: Temperate Grasslands, Savannas and Shrublands; TSCF: Tropical and Subtropical Coniferous Forests; TSDBF: Tropical and Subtropical Dry Broadleaf Forests; TSGSS: Tropical and Subtropical Grasslands, Savannas and Shrublands; TSMBF: Tropical and Subtropical Moist Broadleaf Forests; TD: Tundra. For freshwater ecoregions: LL: Large Lakes; LRD: Large River Deltas; MF: Montane Freshwaters; OI: Oceanic Islands; PF: Polar Freshwaters; TCR: Temperate Coastal Rivers; TFRW: Temperate Floodplain Rivers and Wetlands; TUR: Temperate Upland Rivers; TSCR: Tropical and Subtropical Coastal Rivers; TSFRWC: Tropical and Subtropical Floodplain Rivers and Wetland Complexes; TSUR: Tropical and Subtropical Upland Rivers; XFEB: Xeric Freshwaters and Endorheic (closed) Basins. [file peerj-07-6479-s003.png]

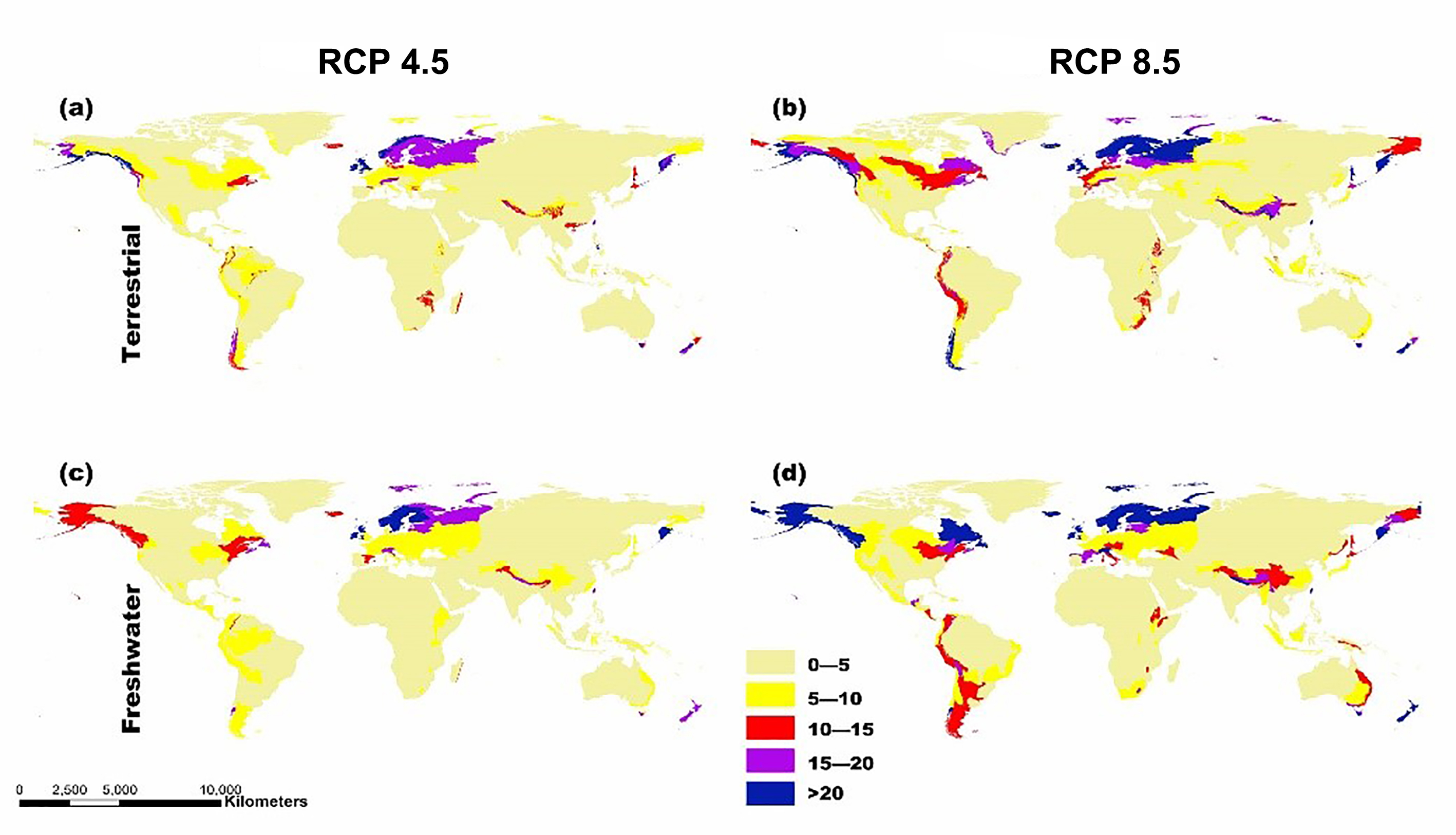

Supplement: Supplemental Information 4 — The colors coupled with the numbers in this figure represent the level of IPS expansion potential across different ecoregions. Blue means there is a very high chance of expansion and tan-yellow means a low chance of expansion. [file peerj-07-6479-s004.png]
